# Estimates of state-level chronic hepatitis C virus infection, stratified by race and sex, United States, 2010

**DOI:** 10.1186/s12879-018-3133-6

**Published:** 2018-05-16

**Authors:** Eric W. Hall, Eli S. Rosenberg, Patrick S. Sullivan

**Affiliations:** 1 Department of Epidemiology, Emory University Rollins School of Public Health, 1518 Clifton Road, GCR 432, Atlanta, GA 30322 USA; 2 Department of Epidemiology and Biostatistics, University at Albany School of Public Health, 1 University Place, rm 123, Rensselaer, NY 12144 USA

**Keywords:** Hepatitis C, Prevalence, NHANES, Mortality, Small-area

## Abstract

**Background:**

Hepatitis C virus (HCV) is the most common blood-borne viral infection in the United States. Previously, we used data from the National Health and Nutrition Examination Survey (NHANES) and mortality data from the National Vital Statistics System (NVSS) to estimate the prevalence of HCV antibodies (anti-HCV) and HCV RNA among all U.S. states. However, demographic differences in HCV burden at the state-level have not been systematically described. This analysis quantified the HCV burden stratified by sex and race (and associated disparities) for each U.S. state.

**Methods:**

Building on our previous method, we used three publicly available data sources to estimate HCV RNA prevalence among noninstitutionalized adults stratified by sex and race group. We used a small-area estimation approach that included direct standardization of NHANES demographic data with logistic regression modeling of HCV-related mortality data as an adjustment factor to estimate the state-level prevalence and total persons with chronic HCV infection for sex and race groups in all U.S. states.

**Results:**

Nationally, males had an estimated HCV RNA prevalence of 1.56% (95% CI: 1.37–1.84%) and females had a prevalence of 0.75% (95% CI: 0.63–0.96%). Stratified by race, national estimated prevalence of HCV RNA was highest among non-Hispanic black (2.43, 95% CI: 2.10–2.90%), followed by non-Hispanic white (1.05, 95% CI: 0.90–1.27%) and Hispanic/other (0.74, 95% CI: 0.59–1.04%). Males in most jurisdictions (41/51) have an HCV RNA prevalence that is between 1.5 and 2.5 times higher than their female counterparts.

**Conclusions:**

HCV infection disparities by sex are mostly consistent across the country. However, race differences in HCV infection differ by state and tailored prevention and treatment efforts specific to the local HCV epidemic are needed to reduce race disparities.

**Electronic supplementary material:**

The online version of this article (10.1186/s12879-018-3133-6) contains supplementary material, which is available to authorized users.

## Background

Hepatitis C virus (HCV) infection is the most common blood-borne infection in the United States [1]. In 2010, an estimated 3.9 million adults had antibodies to HCV (anti-HCV), indicating a previous or current acute infection [2]. Approximately 26% of individuals with acute HCV spontaneously clear infection within 6 months of exposure, but the remaining persons develop a chronic infection [3]. Despite the recent development of curative therapies, chronic HCV remains a leading cause of hepatocellular carcinoma, cirrhosis and liver failure requiring a transplant [4, 5]. Chronic HCV infection has been associated with an increase in HCV-related mortality and in 2007, chronic HCV surpassed HIV as a cause of death in the United States [6, 7].

Current national surveillance efforts provide an incomplete picture of the burden of both HCV infection and diagnoses in the United States. Both acute and chronic HCV infection diagnoses are reportable conditions in the Centers for Disease Control and Prevention (CDC) National Notifiable Disease Surveillance System (NNDSS) [8]. However, many HCV infections are not captured and surveillance data do not accurately represent the burden of hepatitis C in the United States [9]. Chronic HCV is often asymptomatic and about half of infected persons are unaware of their infection, and therefore could not be reported to surveillance systems [10]. Furthermore, some states do not submit viral hepatitis reports to NNDSS (only 37 states reported HCV cases in 2014) and some states report implausibly low numbers [9]. In 2015, CDC directly funded enhanced case surveillance activities in 8 jurisdictions [11]. Both the O’Neill Institute and the US National Viral Hepatitis Action Plan (Goals 4.2 and 4.3) calls for improvements in mechanisms and timeliness of data to monitor the epidemic [12, 13]. In the absence of complete surveillance data, probability-based surveys, such as the National Health and Nutrition Examination Survey (NHANES), can be used to fill this gap, for estimating HCV prevalence on a national level and for the incorporation into our recent model that estimated HCV prevalence at the state-level using a small-area estimation approach [2, 14, 15].

Nationally, the prevalence and risk of new HCV infection differs across several key demographic groups. About 70% of all chronic infections are among individuals who were born between 1945 and 1965 [1]. Overall, the prevalence of anti-HCV among adults from the 1945–1965 birth cohort was estimated to be 3.5% in 2010 [1]. NHANES analyses have demonstrated a national prevalence of anti-HCV that is higher in men (1.9% compared vs. 1.1% in women) and non-Hispanic blacks (2.2% vs. 1.3% among non-Hispanic whites) [1]. Disparities by race have been demonstrated repeatedly in programmatic data and in jurisdiction-specific epidemiologic profiles [16], which collectively form the basis for national goals of reducing HCV-related mortality among African-Americans and American Indians/Alaska Natives [12]. However, extant published demographic differences in HCV burden at local levels are not typically described by prevalence indicators (anti-HCV or HCV RNA) and have not been systematically described for all states. This analysis extends our earlier method for state-level prevalence estimation to quantify the burden of chronic hepatitis C infection, stratified by race and by sex in each U.S. state.

## Methods

### Data sources

We extended a previously-described small-area estimation approach that synthesizes NHANES national estimates of HCV infection with state-level data on HCV-related mortality from the National Vital Statistics System [2]. The data sources and analytic methods are briefly described below.

NHANES uses a complex, multistage sampling design to collect nationally representative questionnaire and laboratory data on the health of the non-institutionalized United States population [17]. Data and corresponding sampling weights are released every 2 years. Data from seven NHANES release cycles (1999–2012) were pooled in order to ensure sufficient data for all demographic groups. All analyses were restricted to respondents aged ≥18 years of age at time of survey. Race/ethnicity was categorized into non-Hispanic black, non-Hispanic white and Hispanic/other. Similar to previous analyses, birth year was classified into three cohorts: before 1945, 1945–1965 and after 1965 [18, 19].

NHANES tested lab samples for anti-HCV using an anti-HCV screening chemiluminescence immunoassay and a confirmatory recombinant immunoblot assay (RIBA) [20]. Samples with positive results on RIBA were tested for HCV RNA using an in vitro nucleic acid amplification test. Participants with a positive or indeterminate anti-HCV test and positive HCV RNA test were considered to represent chronic HCV infection. As done in previous analyses, participants who tested positive for anti-HCV but negative for HCV RNA (resolved infections; *n* = 112) and those who tested positive for anti-HCV but did not have a HCV RNA test (*n* = 160) were not included in this analysis [14].

The National Vital Statistics System (NVSS) collects demographic, geographic and cause of death data from all death certificates in the United States [21]. Mortality Multiple Cause Microdata files (from 1999 to 2012) were used and data were categorized into the same sex, race and birth cohort categories described above. Any death records that listed the ICD-10 code for acute viral hepatitis C (B17.1) or chronic viral hepatitis C (B18.2) as the underlying or a multiple cause of death were considered to indicate HCV-related mortality.

Annual intercensal population estimates (1999–2012) from the US Vintage 2000, 2009 and 2014 data sets were used as denominators for HCV-related mortality rates within each demographic strata [22]. Microdata from the 2010 5-year American Community Survey (ACS, years 2006–2010) were used to generate estimates of the non-institutionalized population [23, 24], which were combined with our estimated number of HCV cases to estimate HCV prevalence rates for each state. The incorporation of ACS population totals into this analysis is an update to our previously published approach and reflect current NHANES guidance [25]. Estimated population total for each sex and race group are reported in Additional file 1: Table S1.

### Analysis

The number of persons with chronic hepatitis C in each state were estimated using a standardization-based estimator described in detail earlier [2]. Briefly, we used NHANES data to calculate weighted estimates for national HCV RNA prevalence for 18 strata of sex (2), race/ethnicity (3) and birth cohort (3) [25]. We multiplied these weighted estimates by 2010 ACS 5-year population estimates for the corresponding demographic stratum for each state to generate crude state-by-stratum totals. We used NVSS mortality data and intercensal population totals to fit a high-order logistic regression model the average HCV-related death rates in the corresponding 12 strata over the 14-year period. We compared observed state-by-strata HCV-related mortality totals to model predictions to assess collinearity and model fit [26, 27]. A ratio of state-by-demographic stratum effects were calculated by comparing state specific HCV-related death rates to national HCV-related death rates (within the same strata). The crude state-by-stratum HCV RNA totals were adjusted by the state-by-stratum effects to calculate mortality-adjusted HCV RNA prevalence totals in each strata in each state. Totals were summed within single strata of race (white non-Hispanic, black non-Hispanic and Hispanic/other race) or sex and divided by corresponding population totals. This yielded HCV RNA prevalence rate estimates by sex and race for each state. Prevalence rate ratios were generated to compare HCV RNA prevalence for men vs women and non-Hispanic black vs non-Hispanic white. An analogous approach and model was used to estimate the prevalence rate and number of persons with HCV antibodies (anti-HCV), which is often used as an indicator of past or current infection [14]. The results for anti-HCV prevalence by race and sex in each state are presented in Additional file 1: Tables S2-S5.

We calculated 95% confidence intervals (CI) for state-level estimates to account for the joint statistical uncertainty in the NHANES prevalence estimates and HCV-related mortality rate estimates from the logistic regression model. Confidence intervals were calculated using a Monte Carlo simulation that sampled from logit-normal and normal distributions, respectively (k = 10,000). We calculated the coefficient of variation for all prevalence rate and ratio estimates. Similar to the guidelines used by NVSS, any estimates with a coefficient of variation of 23% or greater are potentially unreliable and marked accordingly [28].

## Results

There were an estimated 2,583,986 (95% CI: 2,338,079–2,978,150) non-institutionalized US adults with chronic HCV infection in 2010, which corresponds to a prevalence of 1.14% (95% CI: 1.03–1.32%). Nationally, males had an HCV RNA prevalence of 1.56% (95% CI: 1.37–1.84%) and females had a prevalence of 0.75% (95% CI: 0.63–0.96%) (Table 1). Stratified by race, prevalence of HCV RNA was highest among non-Hispanic black (2.43, 95% CI: 2.10–2.90%), followed by non-Hispanic white (1.05, 95% CI: 0.90–1.27%) and Hispanic/other (0.74, 95% CI: 0.59–1.04%) (Tables 2, 3 and 4).

**Table 1:** Estimated total and prevalence rate with chronic hepatitis C infection by sex, US States and District of Columbia, 2010^a^

	Female				Male						
	HCV RNA prevalence rate (per 100)		Total persons^b^ with HCV RNA		HCV RNA Prevalence rate (per 100)			Total persons with HCV RNA		Rate ratio (ref = Female)	
State	Rate	(95% CI)	n	(95% CI)	Rate	(95% CI)		n	(95% CI)	Ratio	(95% CI)
ALABAMA	0.69	(0.57 0.89)	12,720	(10,481 16,461)	1.36	(1.17 1.62)		22,615	(19,448 26,975)	1.98	(1.48 2.57)
ALASKA	1.21	(0.89 1.87)	2915	(2,133 4,502)	1.49	(1.22 1.92)		3,859	(3,164 4,968)	1.23	(0.79 1.81)
ARIZONA	0.75	(0.60 1.02)	17,505	(14,091 23,769)	1.75	(1.49 2.10)		38,770	(33,121 46,690)	2.33	(1.65 3.10)
ARKANSAS	0.69	(0.55 0.94)	7645	(6,094 10,384)	1.64	(1.38 1.99)		16,652	(14,001 20,215)	2.36	(1.67 3.20)
CALIFORNIA	1.04	(0.86 1.39)	143,193	(118,480 190,894)	2.04	(1.78 2.42)		268,277	(234,254 318,754)	1.96	(1.42 2.54)
COLORADO	0.75	(0.61 1.01)	13,846	(11,246 18,633)	1.59	(1.37 1.90)		28,476	(24,518 34,108)	2.11	(1.51 2.78)
CONNECTICUT	0.57	(0.48 0.75)	7974	(6,681 10,408)	1.30	(1.13 1.55)		16,562	(14,390 19,681)	2.28	(1.70 2.91)
DELAWARE	0.94	(0.76 1.25)	3,279	(2,664 4,375)	1.93	(1.65 2.36)		6,086	(5,197 7,437)	2.05	(1.49 2.75)
DISTRICT OF COLUMBIA	2.40	(1.87 3.20)	6,116	(4,771 8,163)	3.12	(2.58 3.92)		6,844	(5,651 8,590)	1.30	(0.92 1.81)
FLORIDA	0.73	(0.60 0.96)	54,329	(44,975 71,090)	1.57	(1.35 1.87)		107,089	(92,016 127,575)	2.15	(1.58 2.79)
GEORGIA	0.55	(0.46 0.71)	19,954	(16,645 25,475)	1.16	(1.00 1.37)		37,754	(32,655 44,598)	2.09	(1.58 2.67)
HAWAII	0.49	(0.37 0.76)	2513	(1,894 3,933)	1.37	(1.16 1.68)		6,937	(5,861 8,524)	2.81	(1.75 3.99)
IDAHO	0.62	(0.47 0.91)	3441	(2,586 5,025)	1.22	(1.00 1.53)		6,572	(5,411 8,257)	1.96	(1.28 2.83)
ILLINOIS	0.36	(0.31 0.47)	17,719	(14,973 22,752)	0.65	(0.57 0.77)		29,683	(25,847 35,021)	1.81	(1.36 2.29)
INDIANA	0.54	(0.44 0.72)	13,275	(10,800 17,573)	1.12	(0.95 1.35)		25,482	(21,695 30,652)	2.06	(1.49 2.73)
IOWA	0.39	(0.30 0.56)	4483	(3,460 6,418)	1.02	(0.86 1.26)		11,227	(9,425 13,871)	2.63	(1.76 3.69)
KANSAS	0.58	(0.46 0.80)	6118	(4,869 8,406)	1.31	(1.10 1.60)		13,131	(11,043 16,037)	2.25	(1.58 3.07)
KENTUCKY	0.66	(0.52 0.92)	11,040	(8,643 15,204)	1.50	(1.25 1.86)		23,080	(19,226 28,571)	2.26	(1.56 3.13)
LOUISIANA	1.10	(0.92 1.40)	18,743	(15,656 23,771)	2.18	(1.90 2.57)		33,463	(29,151 39,519)	1.98	(1.51 2.53)
MAINE	0.33	(0.24 0.51)	1762	(1,262 2,717)	1.12	(0.91 1.44)		5,602	(4,527 7,177)	3.42^c^	(2.11 5.22)
MARYLAND	1.05	(0.87 1.34)	23,576	(19,653 30,142)	1.72	(1.50 2.02)		34,714	(30,318 40,706)	1.65	(1.25 2.10)
MASSACHUSETTS	0.60	(0.49 0.81)	15,784	(12,882 21,165)	1.35	(1.16 1.61)		31,749	(27,376 38,029)	2.22	(1.60 2.92)
MICHIGAN	0.65	(0.54 0.82)	25,033	(20,984 31,617)	1.28	(1.11 1.51)		45,682	(39,723 53,804)	1.97	(1.50 2.50)
MINNESOTA	0.42	(0.35 0.55)	8333	(6,876 10,999)	0.99	(0.85 1.18)		18,974	(16,364 22,647)	2.36	(1.74 3.08)
MISSISSIPPI	0.71	(0.58 0.93)	8034	(6,574 10,554)	1.56	(1.33 1.89)		15,610	(13,332 18,890)	2.19	(1.60 2.87)
MISSOURI	0.72	(0.59 0.95)	16,628	(13,620 21,803)	1.62	(1.39 1.93)		34,139	(29,234 40,812)	2.23	(1.64 2.93)
MONTANA	0.88	(0.66 1.30)	3298	(2,476 4,860)	1.46	(1.21 1.85)		5,361	(4,433 6,798)	1.65	(1.08 2.40)
NEBRASKA	0.52	(0.42 0.72)	3535	(2,827 4,895)	1.06	(0.90 1.31)		6,883	(5,833 8,491)	2.03	(1.42 2.77)
NEVADA	0.69	(0.55 0.94)	6725	(5,392 9,135)	1.74	(1.47 2.11)		16,979	(14,362 20,621)	2.52	(1.77 3.38)
NEW HAMPSHIRE	0.38	(0.28 0.59)	1970	(1,421 3,026)	1.06	(0.86 1.35)		5,168	(4,193 6,630)	2.76	(1.71 4.19)
NEW JERSEY	0.65	(0.55 0.83)	22,188	(18,789 28,416)	1.26	(1.10 1.48)		39,331	(34,413 46,239)	1.95	(1.48 2.46)
NEW MEXICO	1.11	(0.85 1.65)	8457	(6,473 12,632)	2.48	(2.07 3.15)		17,671	(14,799 22,460)	2.23	(1.44 3.20)
NEW YORK	0.74	(0.63 0.95)	57,238	(48,850 73,330)	1.42	(1.26 1.66)		98,479	(87,408 114,701)	1.92	(1.46 2.38)
NORTH CAROLINA	0.71	(0.59 0.90)	25,610	(21,296 32,564)	1.62	(1.41 1.91)		53,569	(46,585 63,012)	2.30	(1.74 2.94)
NORTH DAKOTA	0.29^c^	(0.20 0.51)	719^c^	(509 1,273)	0.74	(0.60 1.01)		1,864	(1,503 2,545)	2.57^c^	(1.40 4.05)
OHIO	0.60	(0.50 0.77)	26,886	(22,401 34,365)	1.33	(1.15 1.57)		54,497	(47,135 64,214)	2.21	(1.67 2.84)
OKLAHOMA	1.51	(1.19 2.07)	21,062	(16,592 28,918)	2.73	(2.31 3.30)		35,554	(30,068 43,031)	1.81	(1.26 2.46)
OREGON	1.27	(0.97 1.76)	18,567	(14,291 25,838)	2.71	(2.27 3.32)		37,859	(31,612 46,314)	2.15	(1.48 3.02)
PENNSYLVANIA	0.62	(0.52 0.78)	31,075	(26,101 39,459)	1.41	(1.22 1.66)		64,388	(55,988 75,877)	2.28	(1.73 2.90)
RHODE ISLAND	0.95	(0.76 1.32)	4052	(3,234 5,641)	1.88	(1.60 2.30)		7,240	(6,149 8,853)	1.98	(1.38 2.69)
SOUTH CAROLINA	0.79	(0.66 1.02)	14,076	(11,646 18,121)	1.73	(1.49 2.05)		27,740	(23,940 32,864)	2.18	(1.63 2.82)
SOUTH DAKOTA	0.39	(0.28 0.63)	1155	(847 1,889)	0.87	(0.70 1.15)		2,502	(2,036 3,317)	2.23^c^	(1.32 3.37)
TENNESSEE	1.05	(0.85 1.38)	25,633	(20,703 33,783)	2.43	(2.06 2.92)		53,969	(45,855 65,023)	2.31	(1.68 3.07)
TEXAS	0.95	(0.80 1.22)	84,617	(71,627 109,034)	1.93	(1.69 2.27)		161,293	(141,235 190,237)	2.03	(1.52 2.58)
UTAH	0.39	(0.30 0.56)	3541	(2,739 5,073)	0.84	(0.70 1.05)		7,497	(6,254 9,351)	2.17	(1.45 3.08)
VERMONT	0.53	(0.37 0.86)	1335	(934 2,136)	1.41	(1.12 1.85)		3,335	(2,640 4,383)	2.64^c^	(1.57 4.16)
VIRGINIA	0.47	(0.40 0.61)	14,500	(12,153 18,560)	1.10	(0.96 1.30)		31,281	(27,183 36,821)	2.33	(1.77 2.96)
WASHINGTON	0.98	(0.78 1.32)	24,627	(19,593 33,172)	2.08	(1.76 2.51)		50,526	(42,851 60,918)	2.12	(1.51 2.88)
WEST VIRGINIA	0.60	(0.45 0.89)	4450	(3,326 6,579)	1.55	(1.27 1.96)		10,674	(8,745 13,462)	2.57	(1.66 3.73)
WISCONSIN	0.31	(0.25 0.40)	6628	(5,467 8,715)	0.70	(0.60 0.83)		14,320	(12,291 17,191)	2.26	(1.66 2.95)
WYOMING	0.83	(0.60 1.31)	1684	(1,220 2,653)	1.44	(1.15 1.92)		2,981	(2,370 3,969)	1.74^c^	(1.04 2.67)
U.S. STATES & WASHINGTON D.C.	0.75	(0.63 0.96)	879,589	(743,338 1,128,362)	1.56	(1.37 1.84)		1,699,989	(1,486,193 2,001,396)	2.08	(1.57 2.63)

*Abbreviations*: CI confidence interval, HCV RNA hepatitis C virus ribonucleic acid

^a^Defined as persons with HCV RNA

^b^Represents the non-institutionalized population

^c^Coefficient of variation is ≥ 23%; estimate is unreliable

**Table 2:** Estimated total and prevalence rate with chronic hepatitis C infection among non-Hispanic white persons, US States and District of Columbia, 2010^a^

	HCV RNA prevalence rate (per 100)		Total persons with HCV RNA^b^	
State	Rate	(95% CI)	n	(95% CI)
ALABAMA	0.94	(0.80 1.14)	23,139	(19,654 28,258)
ALASKA	1.54	(1.27 2.01)	5285	(4,332 6,869)
ARIZONA	1.35	(1.15 1.64)	39,779	(33,842 48,151)
ARKANSAS	1.15	(0.97 1.41)	19,102	(16,089 23,463)
CALIFORNIA	1.88	(1.61 2.27)	231,304	(197,678 278,733)
COLORADO	1.02	(0.86 1.24)	27,578	(23,299 33,469)
CONNECTICUT	0.72	(0.61 0.88)	14,426	(12,215 17,645)
DELAWARE	1.09	(0.91 1.37)	5078	(4,232 6,410)
DISTRICT OF COLUMBIA	0.43	(0.34 0.61)	770	(605 1,073)
FLORIDA	1.29	(1.10 1.56)	115,222	(98,297 139,057)
GEORGIA	0.82	(0.70 1.00)	34,005	(28,820 41,321)
HAWAII	2.29	(1.90 2.87)	6043	(5,014 7,568)
IDAHO	0.93	(0.78 1.16)	8838	(7,399 11,049)
ILLINOIS	0.44	(0.37 0.53)	28,061	(23,875 34,244)
INDIANA	0.72	(0.61 0.88)	28,747	(24,444 35,069)
IOWA	0.64	(0.54 0.80)	13,279	(11,198 16,460)
KANSAS	0.89	(0.75 1.10)	15,080	(12,680 18,563)
KENTUCKY	1.00	(0.83 1.23)	28,246	(23,690 34,890)
LOUISIANA	1.30	(1.10 1.58)	27,096	(23,024 33,040)
MAINE	0.71	(0.59 0.90)	7033	(5,856 8,877)
MARYLAND	0.92	(0.78 1.13)	22,931	(19,392 28,097)
MASSACHUSETTS	0.85	(0.72 1.03)	33,610	(28,529 40,907)
MICHIGAN	0.69	(0.59 0.84)	41,050	(34,774 49,679)
MINNESOTA	0.54	(0.45 0.65)	18,190	(15,369 22,162)
MISSISSIPPI	1.20	(1.01 1.48)	15,857	(13,373 19,566)
MISSOURI	0.98	(0.84 1.20)	36,273	(30,840 44,098)
MONTANA	1.07	(0.88 1.35)	7135	(5,907 9,051)
NEBRASKA	0.66	(0.55 0.82)	7474	(6,231 9,366)
NEVADA	1.54	(1.30 1.89)	18,219	(15,334 22,295)
NEW HAMPSHIRE	0.71	(0.59 0.90)	6719	(5,600 8,491)
NEW JERSEY	0.86	(0.73 1.04)	35,420	(30,164 43,063)
NEW MEXICO	1.69	(1.43 2.07)	11,576	(9,752 14,142)
NEW YORK	0.73	(0.62 0.88)	65,307	(55,785 78,812)
NORTH CAROLINA	0.99	(0.84 1.19)	47,365	(40,211 57,252)
NORTH DAKOTA	0.45	(0.36 0.63)	2084	(1,671 2,883)
OHIO	0.72	(0.61 0.87)	51,804	(43,989 62,811)
OKLAHOMA	2.31	(1.96 2.82)	46,062	(39,054 56,331)
OREGON	2.11	(1.79 2.56)	50,091	(42,511 60,837)
PENNSYLVANIA	0.74	(0.63 0.89)	58,578	(49,866 70,820)
RHODE ISLAND	1.21	(1.01 1.52)	7,962	(6,652 9,982)
SOUTH CAROLINA	1.15	(0.97 1.40)	26,141	(22,134 31,835)
SOUTH DAKOTA	0.54	(0.43 0.72)	2793	(2,243 3,768)
TENNESSEE	1.63	(1.38 1.99)	59,986	(50,921 73,192)
TEXAS	1.51	(1.29 1.82)	132,698	(113,228 160,163)
UTAH	0.60	(0.50 0.75)	9051	(7,587 11,269)
VERMONT	0.96	(0.79 1.25)	4460	(3,664 5,782)
VIRGINIA	0.65	(0.55 0.79)	26,111	(22,183 31,786)
WASHINGTON	1.58	(1.34 1.91)	60,245	(51,196 72,808)
WEST VIRGINIA	1.01	(0.84 1.27)	13,625	(11,335 17,068)
WISCONSIN	0.39	(0.33 0.47)	14,229	(12,029 17,463)
WYOMING	1.12	(0.91 1.48)	4041	(3,287 5,336)
U.S. STATES & WASHINGTON D.C.	1.05	(0.90 1.27)	1,615,202	(1,380,727 1,947,882)

*Abbreviations*: *CI* confidence interval, *HCV RNA* hepatitis C virus ribonucleic acid

^a^Defined as persons with HCV RNA

^b^Represents the non-institutionalized population

**Table 3:** Estimated total and prevalence rate with chronic hepatitis C infection among non-Hispanic black persons, US States and District of Columbia, 2010^a^

	HCV RNA prevalence rate (per 100)		Total persons with HCV RNA^b^		Rate ratio (ref = white)		
State	Rate	(95% CI)	n	(95% CI)	Ratio	(95% CI)	
ALABAMA	1.39	(1.19 1.69)	11,817	(10,098 14,362)	1.48	(1.15 1.90)	
ALASKA	2.27	(1.64 3.72)	352	(255 579)	1.47	(1.06 2.32)	
ARIZONA	2.42	(2.03 2.99)	3732	(3,129 4,606)	1.79	(1.39 2.33)	
ARKANSAS	1.65	(1.39 2.03)	4863	(4,113 6,010)	1.43	(1.10 1.87)	
CALIFORNIA	4.12	(3.56 4.91)	63,293	(54,696 75,368)	2.19	(1.73 2.75)	
COLORADO	3.70	(3.14 4.51)	4604	(3,897 5,599)	3.64	(2.81 4.69)	
CONNECTICUT	2.65	(2.24 3.24)	6003	(5,086 7,339)	3.70	(2.86 4.78)	
DELAWARE	3.21	(2.69 4.03)	4009	(3,354 5,036)	2.95	(2.21 3.95)	
DISTRICT OF COLUMBIA	5.23	(4.45 6.41)	11,997	(10,209 14,709)	12.03	(8.36 16.51)	
FLORIDA	1.54	(1.33 1.85)	29,046	(25,019 34,879)	1.20	(0.94 1.52)	
GEORGIA	1.17	(1.00 1.41)	22,519	(19,281 27,139)	1.42	(1.11 1.81)	
HAWAII	2.29	(1.73 3.32)	352	(266 510)	1.00	(0.70 1.49)	
IDAHO	1.37^c^	(0.77 3.41)	70^c^	(40 174)	1.48^c^	(0.79 3.71)	
ILLINOIS	1.28	(1.10 1.54)	15,988	(13,747 19,242)	2.91	(2.28 3.70)	
INDIANA	2.43	(2.08 2.94)	9022	(7,728 10,951)	3.38	(2.63 4.33)	
IOWA	3.31	(2.70 4.26)	1644	(1,343 2,116)	5.13	(3.85 6.93)	
KANSAS	2.62	(2.20 3.26)	2790	(2,334 3,465)	2.94	(2.25 3.86)	
KENTUCKY	2.46	(2.07 3.07)	5442	(4,573 6,771)	2.48	(1.89 3.25)	
LOUISIANA	2.60	(2.23 3.14)	24,261	(20,834 29,291)	2.00	(1.562.56)	
MAINE	2.30^c^	(1.55 3.95)	169^c^	(114 291)	3.24^c^	(2.02 5.70)	
MARYLAND	2.90	(2.48 3.50)	33,960	(29,082 41,053)	3.15	(2.45 4.05)	
MASSACHUSETTS	2.84	(2.42 3.47)	7788	(6,626 9,510)	3.36	(2.60 4.32)	
MICHIGAN	2.82	(2.43 3.38)	26,795	(23,037 32,127)	4.06	(3.18 5.15)	
MINNESOTA	4.10	(3.46 5.03)	6371	(5,367 7,809)	7.66	(5.91 9.89)	
MISSISSIPPI	1.03	(0.88 1.27)	7470	(6,358 9,183)	0.86	(0.66 1.12)	
MISSOURI	2.94	(2.52 3.54)	13,252	(11,367 15,967)	2.98	(2.33 3.82)	
MONTANA	5.04^c^	(3.29 9.29)	127^c^	(83 235)	4.72^c^	(2.87 8.82)	
NEBRASKA	4.16	(3.44 5.34)	2132	(1,763 2,733)	6.36	(4.74 8.63)	
NEVADA	1.90	(1.59 2.35)	2626	(2,200 3,245)	1.23	(0.94 1.60)	
NEW HAMPSHIRE	2.07^c^	(1.39 3.49)	186^c^	(126 315)	2.90^c^	(1.82 5.00)	
NEW JERSEY	2.44	(2.11 2.94)	19,250	(16,610 23,150)	2.85	(2.23 3.62)	
NEW MEXICO	2.66	(2.12 3.54)	685	(545 911)	1.57	(1.16 2.18)	
NEW YORK	2.76	(2.39 3.29)	55,406	(47,847 65,884)	3.80	(3.00 4.80)	
NORTH CAROLINA	2.14	(1.84 2.57)	29,472	(25,328 35,455)	2.17	(1.70 2.76)	
NORTH DAKOTA	0.83^c^	(0.44 2.67)	39^c^	(20 125)	1.82^c^	(0.87 5.93)	
OHIO	2.99	(2.58 3.58)	27,791	(23,967 33,281)	4.16	(3.26 5.27)	
OKLAHOMA	2.61	(2.21 3.21)	4594	(3,883 5,637)	1.13	(0.87 1.47)	
OREGON	4.93	(4.10 6.13)	2092	(1,742 2,603)	2.33	(1.78 3.05)	
PENNSYLVANIA	3.51	(3.02 4.21)	30,986	(26,683 37,175)	4.77	(3.75 6.04)	
RHODE ISLAND	5.32	(4.39 6.83)	1979	(1,634 2,540)	4.39	(3.27 5.90)	
SOUTH CAROLINA	1.69	(1.45 2.04)	14,899	(12,752 17,960)	1.48	(1.15 1.88)	
SOUTH DAKOTA	3.16^c^	(2.10 5.85)	147^c^	(97 272)	5.89^c^	(3.50 11.16)	
TENNESSEE	2.66	(2.27 3.22)	18,656	(15,900 22,622)	1.63	(1.26 2.10)	
TEXAS	3.10	(2.66 3.72)	58,916	(50,662 70,870)	2.05	(1.61 2.60)	
UTAH	2.60	(1.92 3.80)	386	(286 564)	4.32	(2.97 6.49)	
VERMONT	2.61^c^	(1.70 4.95)	90^c^	(58 170)	2.72^c^	(1.64 5.26)	
VIRGINIA	1.66	(1.42 2.00)	17,734	(15,209 21,337)	2.56	(2.00 3.27)	
WASHINGTON	4.41	(3.77 5.34)	6780	(5,794 8,214)	2.79	(2.19 3.58)	
WEST VIRGINIA	3.60	(2.95 4.62)	1434	(1,175 1,840)	3.55	(2.63 4.82)	
WISCONSIN	2.55	(2.16 3.11)	5372	(4,555 6,568)	6.59	(5.07 8.52)	
WYOMING	1.88^c^	(1.12 4.47)	53^c^	(32 126)	1.68^c^	(0.92 4.01)	
U.S. STATES & WASHINGTON D.C.	2.43	(2.10 2.90)	619,438	(536,458 739,838)	2.31	(1.83 2.91)	

*Abbreviations*: *CI* confidence interval, *HCV RNA* hepatitis C virus ribonucleic acid

^a^Defined as persons with HCV RNA

^b^Represents the non-institutionalized population

^c^Coefficient of variation is ≥ 23%; estimate is unreliable

**Table 4:** Estimated total and prevalence rate with chronic hepatitis C infection among Hispanic persons, US States and District of Columbia, 2010^a^

	HCV RNA prevalence rate (per 100)		Total persons with HCV RNA^b^		Rate ratio (ref = white)	
State	Rate	(95% CI)	n	(95% CI)	Ratio	(95% CI)
ALABAMA	0.20	(0.15 0.30)	379	(286 573)	0.21	(0.15 0.33)
ALASKA	0.80	(0.59 1.27)	1137	(838 1,805)	0.52	(0.38 0.79)
ARIZONA	0.88	(0.69 1.22)	12,765	(10,112 17,836)	0.65	(0.48 0.94)
ARKANSAS	0.20	(0.15 0.31)	332	(246 512)	0.18	(0.12 0.28)
CALIFORNIA	0.89	(0.71 1.26)	116,873	(92,893 165,162)	0.47	(0.35 0.69)
COLORADO	1.28	(1.02 1.78)	10,139	(8,066 14,121)	1.25	(0.93 1.83)
CONNECTICUT	0.96	(0.75 1.37)	4107	(3,220 5,862)	1.34	(0.99 1.98)
DELAWARE	0.38	(0.28 0.62)	279	(203 446)	0.35^c^	(0.24 0.58)
DISTRICT OF COLUMBIA	0.29	(0.21 0.47)	193	(140 315)	0.66^c^	(0.42 1.13)
FLORIDA	0.50	(0.39 0.71)	17,151	(13,604 24,428)	0.39	(0.29 0.56)
GEORGIA	0.15	(0.11 0.21)	1185	(918 1,718)	0.18	(0.13 0.27)
HAWAII	0.41	(0.32 0.61)	3055	(2,359 4,502)	0.18	(0.13 0.28)
IDAHO	0.83	(0.65 1.21)	1106	(858 1,612)	0.90	(0.65 1.37)
ILLINOIS	0.19	(0.15 0.28)	3352	(2,623 4,950)	0.43	(0.32 0.65)
INDIANA	0.28	(0.22 0.42)	988	(765 1,447)	0.40	(0.29 0.59)
IOWA	0.56	(0.43 0.85)	787	(596 1,190)	0.88	(0.62 1.37)
KANSAS	0.53	(0.41 0.78)	1379	(1,063 2,012)	0.60	(0.43 0.90)
KENTUCKY	0.31	(0.23 0.50)	432	(315 692)	0.31	(0.21 0.51)
LOUISIANA	0.39	(0.30 0.57)	849	(659 1,245)	0.30	(0.22 0.45)
MAINE	0.46	(0.32 0.75)	161	(114 265)	0.64^c^	(0.42 1.09)
MARYLAND	0.23	(0.18 0.34)	1399	(1,091 2,045)	0.25	(0.18 0.38)
MASSACHUSETTS	0.84	(0.66 1.20)	6134	(4,814 8,734)	0.99	(0.73 1.47)
MICHIGAN	0.51	(0.41 0.72)	2869	(2,270 4,036)	0.74	(0.54 1.09)
MINNESOTA	0.77	(0.60 1.09)	2746	(2,154 3,874)	1.44	(1.06 2.10)
MISSISSIPPI	0.37	(0.27 0.57)	318	(237 499)	0.30	(0.21 0.49)
MISSOURI	0.46	(0.36 0.66)	1242	(969 1,780)	0.47	(0.34 0.70)
MONTANA	2.03	(1.56 3.00)	1397	(1,076 2,064)	1.90	(1.35 2.92)
NEBRASKA	0.62	(0.47 0.95)	812	(615 1,243)	0.95	(0.66 1.49)
NEVADA	0.45	(0.36 0.64)	2859	(2,248 4,039)	0.29	(0.22 0.43)
NEW HAMPSHIRE	0.45	(0.32 0.72)	233	(168 373)	0.63^c^	(0.42 1.04)
NEW JERSEY	0.42	(0.33 0.60)	6848	(5,440 9,714)	0.49	(0.37 0.72)
NEW MEXICO	1.81	(1.43 2.56)	13,867	(10,982 19,648)	1.07	(0.79 1.57)
NEW YORK	0.96	(0.77 1.37)	35,004	(27,847 49,662)	1.33	(0.99 1.94)
NORTH CAROLINA	0.32	(0.25 0.46)	2343	(1,812 3,362)	0.32	(0.24 0.48)
NORTH DAKOTA	1.21	(0.89 2.01)	459	(337 766)	2.66^c^	(1.72 4.57)
OHIO	0.40	(0.32 0.57)	1788	(1,413 2,518)	0.56	(0.41 0.81)
OKLAHOMA	1.13	(0.89 1.60)	5960	(4,690 8,439)	0.49	(0.36 0.72)
OREGON	0.95	(0.75 1.32)	4244	(3,343 5,896)	0.45	(0.33 0.65)
PENNSYLVANIA	0.79	(0.62 1.11)	5899	(4,660 8,303)	1.07	(0.79 1.56)
RHODE ISLAND	1.14	(0.88 1.69)	1351	(1,043 2,000)	0.94	(0.67 1.44)
SOUTH CAROLINA	0.36	(0.28 0.52)	775	(600 1,138)	0.31	(0.23 0.47)
SOUTH DAKOTA	1.18	(0.88 1.87)	718	(534 1,140)	2.19^c^	(1.45 3.64)
TENNESSEE	0.34	(0.26 0.51)	960	(727 1,453)	0.21	(0.15 0.32)
TEXAS	0.83	(0.65 1.16)	54,296	(43,036 76,317)	0.55	(0.41 0.80)
UTAH	0.56	(0.44 0.82)	1601	(1,244 2,328)	0.94	(0.68 1.43)
VERMONT	0.64^c^	(0.44 1.13)	120^c^	(83 211)	0.67^c^	(0.42 1.20)
VIRGINIA	0.24	(0.19 0.34)	1935	(1,519 2,749)	0.37	(0.27 0.55)
WASHINGTON	0.84	(0.67 1.18)	8127	(6,478 11,442)	0.53	(0.39 0.77)
WEST VIRGINIA	0.17^c^	(0.11 0.34)	66^c^	(42 134)	0.17^c^	(0.10 0.35)
WISCONSIN	0.41	(0.32 0.59)	1346	(1,047 1,943)	1.05	(0.76 1.57)
WYOMING	1.27	(0.94 2.01)	571	(422 906)	1.13^c^	(0.76 1.86)
U.S. STATES & WASHINGTON D.C.	0.74	(0.59 1.04)	344,938	(275,446 485,020)	0.70	(0.53 1.03)

*Abbreviations*: *CI* confidence interval, *HCV RNA* hepatitis C virus ribonucleic acid

^a^Defined as persons with HCV RNA

^b^Represents the non-institutionalized population

^c^Coefficient of variation is ≥ 23%; estimate is unreliable

**Fig. 1:**
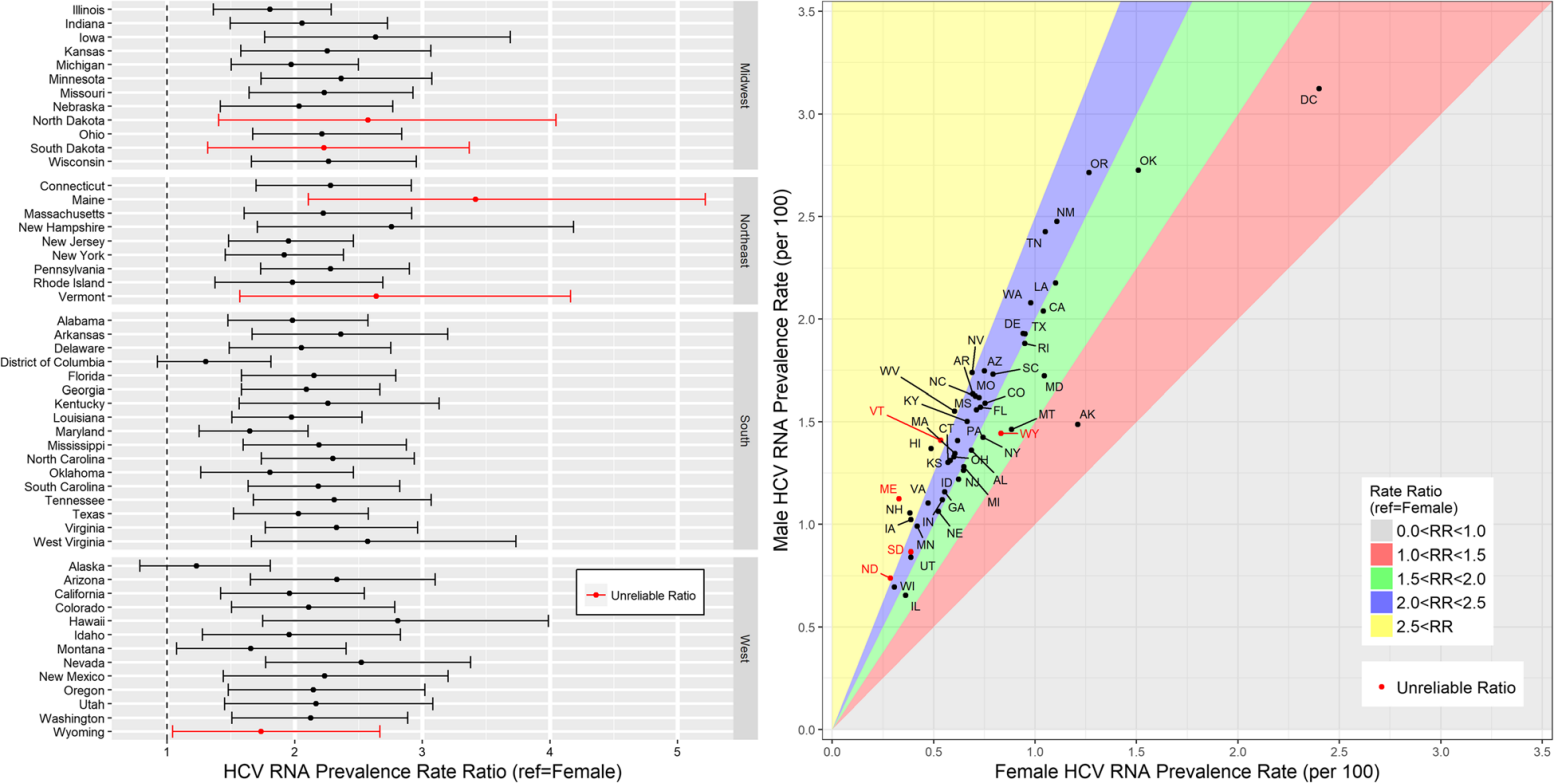
Estimated Prevalence Rate and Rate Ratios of Chronic HCV Infection by Sex, US States and District of Columbia, 2010^a,b^. Abbreviations: HCV RNA, hepatitis C virus ribonucleic acid; RR, rate ratio. ^a^Defined as persons with HCV RNA. ^b^Using estimates of the non-institutionalized population from the 2006–2010 American Community Survey (ACS) 5-year dataset. *Coefficient of variation is ≥ 23%; estimate is unreliable

Among males, estimated HCV RNA prevalence ranged from 0.65% (Illinois) to 3.12% (District of Columbia, Table 1). Among females, reliable prevalence estimates ranged from 0.31% (Wisconsin) to 2.40% (District of Columbia). In all 51 jurisdictions, estimated HCV RNA prevalence was higher in males compared to females (Rate Ratio (RR) > 1, Fig. 1). In all but two jurisdictions (Alaska and District of Columbia) the corresponding rate ratio 95% confidence interval did not include the null value (RR = 1). Of the 51 jurisdictions, 41 have an estimated male-to-female prevalence ratio between 1.5 and 2.5.

**Fig. 2:**
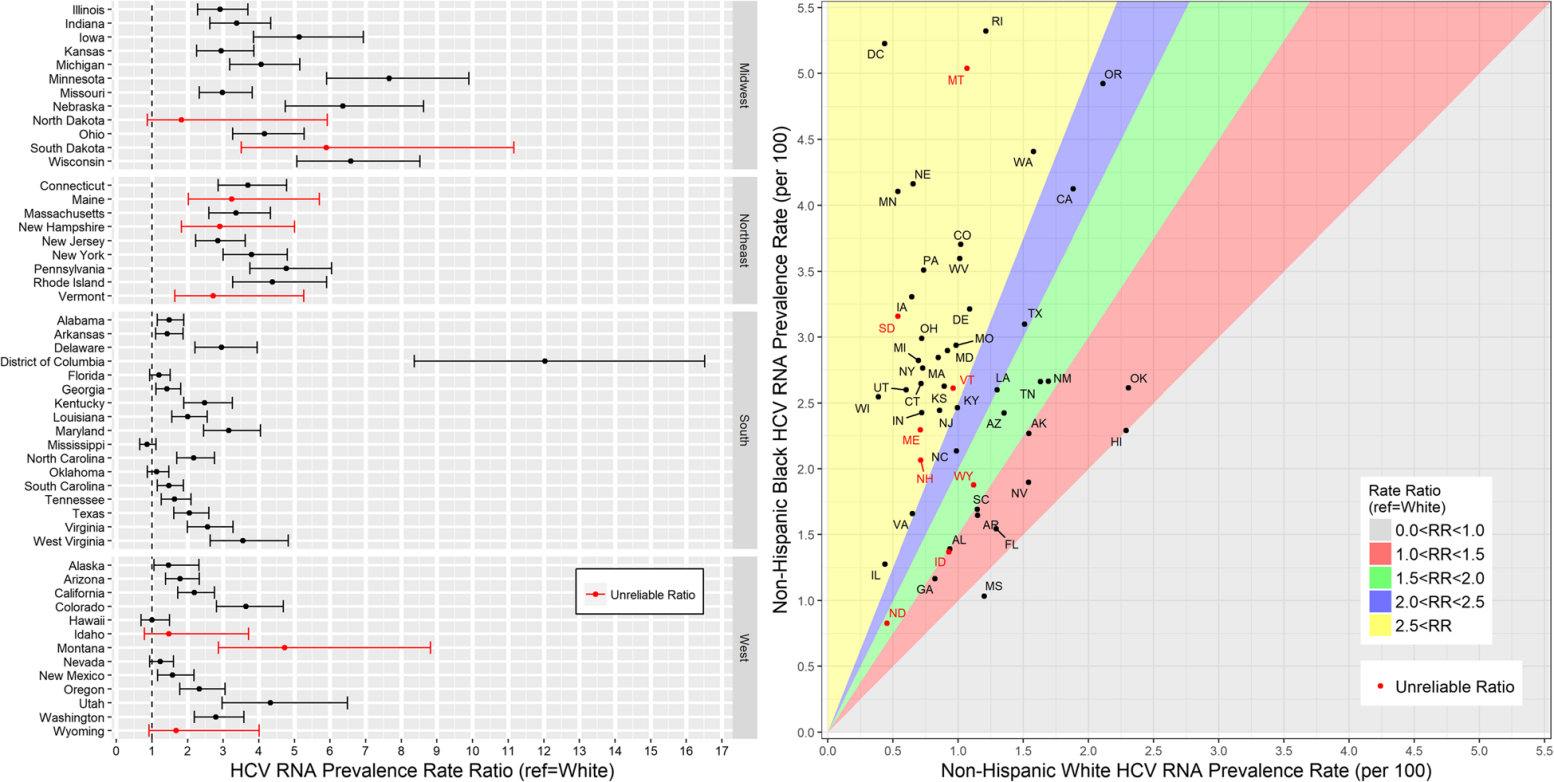
Estimated Prevalence Rate and Rate Ratios of Chronic HCV Infection by non-Hispanic Black and non-Hispanic White Race, US States and District of Columbia, 2010^a,b^. Abbreviations: HCV RNA, hepatitis C virus ribonucleic acid; RR, rate ratio. ^a^Defined as persons with HCV RNA. ^b^Using estimates of the non-institutionalized population from the 2006–2010 American Community Survey (ACS) 5-year dataset. *Coefficient of variation is ≥ 23%; estimate is unreliable

**Fig. 3:**
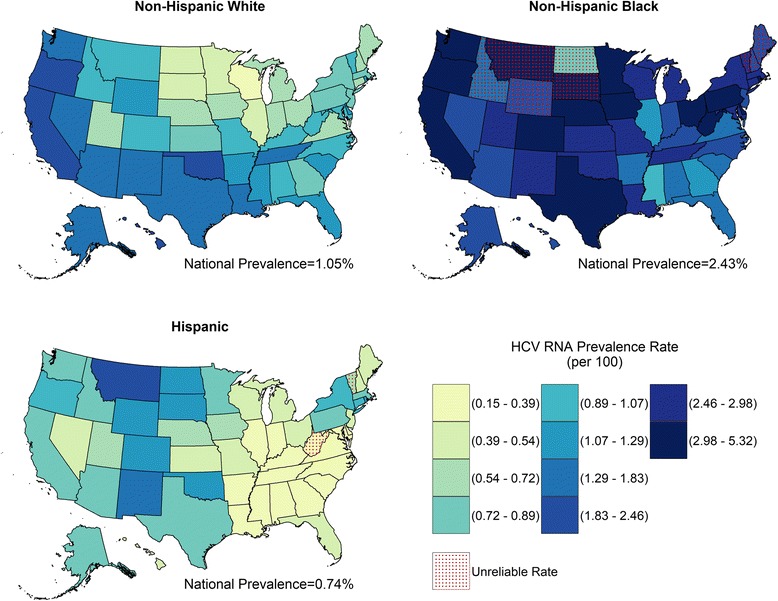
Estimated Prevalence Rate of Chronic HCV Infection by Race, US States and District of Columbia, 2010^a,b^. Abbreviations: HCV RNA, hepatitis C virus ribonucleic acid. ^a^Defined as persons with HCV RNA. ^b^Using estimates of the non-institutionalized population from the 2006–2010 American Community Survey (ACS) 5-year dataset. *Coefficient of variation is ≥ 23%; estimate is unreliable

State-level HCV RNA prevalence among non-Hispanic white persons ranged from 0.39% (Wisconsin) to 2.31% (Oklahoma, Table 2). Prevalence ranged from 0.83% (North Dakota, unreliable estimate) to 5.32 (Rhode Island) among non-Hispanic black persons and from 0.15% (Georgia) to 2.03% (Montana) among Hispanic/other persons (Tables 3 and 4). In all jurisdictions except for Mississippi and Hawaii, the estimated HCV RNA prevalence was higher among non-Hispanic black compared to non-Hispanic white (RR range from 1.13 to 12.03, Fig. 2). The District of Columbia had the largest relative difference in prevalence between non-Hispanic black and non-Hispanic white (RR = 12.03, 95% CI: 8.36–16.51). Race stratified prevalence estimates are displayed geographically in Fig. 3. Among all state-level race-stratified HCV RNA prevalence estimates, the entire top two deciles are from the non-Hispanic black race (Fig. 3).

## Discussion

These results are consistent with previous analysis of chronic HCV infection across demographic groups and provide a more detailed picture of the HCV burden in each US state. Using and NHANES based approach, Denniston et al. estimated a higher prevalence of chronic HCV in men compared to women, which is consistent with all our individual state-level estimates [14]. In general, these results indicate a homogeneity in disparity of HCV prevalence in males and females across the country; with males having roughly double the prevalence as females. This consistency across states is not seen when comparing prevalence of HCV infection by race.

Differences in chronic HCV prevalence by subgroups can be a result of differences in risk behaviors, biological mechanisms, and access to screening and treatment. A higher proportion of men report having ever injected drugs (3.6% vs. 1.6% for women), which is a risk factor for infection [29]. However, female injection drug users have a higher risk of HCV infection than male counterparts [30]. Additionally, the consistent difference in HCV prevalence in men and women is likely the result of a biological mechanism in HCV infection and viral clearance. Spontaneous acute HCV resolution has been shown to be higher in females (40%) compared to males (19%) [3, 31]. The exact mechanism for higher viral clearance in women is unknown, but has been hypothesized to be due to the estrogen hormone [3].

In contrast to the homogeneity seen in the stratified sex estimates, these results indicate racial disparities in HCV infection differ by state. For example, while the estimated HCV RNA prevalence among non-Hispanic whites is similar in Rhode Island (1.21%) and Arkansas (1.15%), the estimated prevalence among non-Hispanic blacks differs greatly between the two states (5.32 and 1.65%, respectively). Even areas with similar relative disparities may have largely different epidemics. California and North Carolina have similar estimated prevalence rate ratios (2.19 and 2.17, respectively) even though prevalence rates are higher across all three race groups in California.

Racial differences in HCV prevalence could result from a variety of societal and biological factors that lead to differences in exposure to risk factors and disease progression. While difficult to assess, state-level differences in racial disparities of transmission risk factors (such as injection drug use) could contribute to differences in HCV prevalence. While the risk of HCV infection during incarceration is not well-know, several surveys indicate that incarcerated individuals have a higher HCV prevalence than the general population [32]. State-level differences in incarceration rates by race might also track with local differences in HCV prevalence. Acute HCV clearance has been hypothesized to be lower in African Americans compared to Hispanic and white counterparts [33–35]. This is supported by viral clearance being lower in genotype 1 infections, which occur more frequently in African Americans. African-Americans are also more likely to be screened for HCV, leading to higher rates of diagnosis [36]. Furthermore, African-Americans, Hispanics and Asians are all less likely to receive traditional treatment (interferon-based) than Caucasians [37, 38]. This is partially because of a higher rate of treatment ineligibility (due to comorbidities) [37, 39] and a lower efficacy for viral genotype 1, which is more common in African Americans [40, 41]. African-Americans are also more likely to defer interferon-based treatment [42–44] and receive direct activing antiretroviral treatment [45]. To address this disparity, CDC has generated a set of materials and public health messages that specifically aim to reduce the HCV burden in the African-American community [46].

### Limitations

This approach uses data from several large, public and population-based data sources. However, there are some limitations to consider when interpreting these results. First, these estimates only represent the non-institutionalized population. Data from NHANES does not represent homeless persons or persons in correctional facilities, nursing homes or other institutions in which chronic HCV prevalence may differ from the general population [47]. Geographic differences in incarceration rates by race and sex could influence statewide HCV prevalence. However, previous work on a national level demonstrates a promising approach for including underrepresented populations that can be extended to the state level in the future [47]. Second, we aggregated 14 years of NHANES data in order to have a sufficient sample size to produce reliable estimates. Slight changes in local HCV incidence over that time period might not be captured in this approach. In the NHANES data, the national overall prevalence of HCV RNA changes slightly from 1999 to 2006 (1.26%; 95% CI: 1.06–1.48%) to 2007–2012 (1.05%; 95%: 0.82–1.34%). However, there is not enough data to reliably determine if there are temporal changes in prevalence within subgroups. Interpreting these results in conjunction with further analysis of local risk behaviors will help state health departments better understand their epidemic. Additionally, we do not present separate results for American Indians/Alaska Natives (AI/AN), who face an elevated HCV burden and are a key population identified in the National Viral Hepatitis Action Plan [12]. Due to under sampling and sparse NHANES data within this group, we are not able to separate AI/AN into an additional race category. We combined AI/AN and other under sampled race groups with Hispanic in order to have sufficient data in each strata of NHANES data. However, 76% of the estimated HCV RNA infections in this combined group were among persons who identified as Hispanic. Similarly, although we control for birth cohort in our model, we do not present results stratified by age. It is challenging to present meaningful estimates for age because we pooled data across 14 years. Finally, despite pooling data, some individual point estimates might still be unreliable. However, those values have been indicated as potentially unreliable, and confidence intervals have been provided for all estimates. Improved direct surveillance data or additional model input data are needed to overcome the demographic limitations of using NHANES data.

## Conclusions

Our approach synthesizes large scale, population based data systems to provide comprehensive state-level estimates that can be used to compare the HCV burden across different states and demographic groups. As the expanded availability of curative therapies continues to increase and acute infection rates rise, this method of prevalence estimation will need to be updated in the future. In the meantime, these data shine an important light on the intersection of geographic and demographic differences of chronic HCV infection. Estimates of chronic HCV prevalence by race and sex can help state health departments understand their local epidemic and assist with their development of targeted prevention and treatment programs. Utilizing these population-based systematic results with local knowledge of HCV risk behaviors and treatment practices provide more granular insight into the chronic hepatitis C epidemic.

## Additional file

Additional file 1: **Tables S1**-**S5** that display population counts used as denominators in rate calculations and HCV antibody prevalence by the same race and sex groups as the primary results. (PDF 1163 kb)

## Abbreviations

ACS: American Community Survey
AI/AN: American Indians/Alaska Natives
anti-HCV: Hepatitis C virus antibodies
CDC: Centers for Disease Control and Prevention
CI: Confidence interval
HCV RNA: Hepatitis C virus ribonucleic acid
HCV: Hepatitis C virus
NHANES: National Health and Nutrition Examination Survey
NNDSS: National Notifiable Disease Surveillance System
NVSS: National Vital Statistics Systems
RIBA: Recombinant immunoblot assay
RR: Rate ratio
U.S.: United States

## References

[1] Ditah I, Ditah F, Devaki P, Ewelukwa O, Ditah C, Njei B, Luma HN, Charlton M (2014). The changing epidemiology of hepatitis C virus infection in the United States: National Health and nutrition examination survey 2001 through 2010. J Hepatol.

[2] Rosenberg ES, Hall EW, Sullivan PS, Sanchez TH, Workowski KA, Ward JW, Holtzman D (2017). Estimation of state-level prevalence of hepatitis C virus infection, US states and District of Columbia, 2010. Clinical infectious diseases: an official publication of the Infectious Diseases Society of America.

[3] Micallef JM, Kaldor JM, Dore GJ (2006). Spontaneous viral clearance following acute hepatitis C infection: a systematic review of longitudinal studies. J Viral Hepat.

[4] Lingala S, Ghany MG (2015). Natural history of hepatitis C. Gastroenterol Clin N Am.

[5] Liver Transplantation [http://www.niddk.nih.gov/health-information/health-topics/liver-disease/liver-transplant/Pages/facts.aspx]. Accessed 11 Nov 2016.

[6] Ly KN, Xing J, Klevens RM, Jiles RB, Ward JW, Holmberg SD (2012). The increasing burden of mortality from viral hepatitis in the United States between 1999 and 2007. Ann Intern Med.

[7] Razavi H, Elkhoury AC, Elbasha E, Estes C, Pasini K, Poynard T, Kumar R (2013). Chronic hepatitis C virus (HCV) disease burden and cost in the United States. Hepatology (Baltimore, Md).

[8] National Notifiable Disease Surveillance System (NNDSS) [http://wwwn.cdc.gov/nndss/]. Accessed 11 Nov 2016.

[9] Viral Hepatitis Surveillance, United States, 2014 [http://www.cdc.gov/hepatitis/statistics/2014surveillance/pdfs/2014hepsurveillancerpt.pdf]. Accessed 11 Nov 2016.

[10] Spradling PR, Rupp L, Moorman AC, Lu M, Teshale EH, Gordon SC, Nakasato C, Boscarino JA, Henkle EM, Nerenz DR (2012). Hepatitis B and C virus infection among 1.2 million persons with access to care: factors associated with testing and infection prevalence. Clin Infect Dis.

[11] Surveillance for Viral Hepatitis - United States, 2015: Summary [https://www.cdc.gov/hepatitis/statistics/2015surveillance/commentary.htm]. Accessed 11 Nov 2016.

[12] National Viral Hepatitis Action Plan 2017–2020 [https://www.hhs.gov/sites/default/files/National%20Viral%20Hepatitis%20Action%20Plan%202017-2020.pdf]. Accessed 11 Nov 2016.

[13] Monitoring the Hepatitis C Epidemic in the United States: What Tools Are Needed to Achieve Elimination [https://www.law.georgetown.edu/oneillinstitute/research/documents/HEPC_Brief_1_PF.pdf]. Accessed 11 Nov 2016.

[14] Denniston MM, Jiles RB, Drobeniuc J, Klevens RM, Ward JW, McQuillan GM, Holmberg SD (2014). Chronic hepatitis C virus infection in the United States, National Health and nutrition examination survey 2003 to 2010. Ann Intern Med.

[15] Armstrong GL, Wasley A, Simard EP, McQuillan GM, Kuhnert WL, Alter MJ (2006). The prevalence of hepatitis C virus infection in the United States, 1999 through 2002. Ann Intern Med.

[16] Hepatitis C Infections in Oregon [http://www.oregon.gov/oha/PH/DISEASESCONDITIONS/HIVSTDVIRALHEPATITIS/ADULTVIRALHEPATITIS/Documents/Hepatitis-C-in-Oregon.pdf]. Accessed 11 Nov 2016.

[17] National Health and Nutrition Examination Survey (NHANES) [http://www.cdc.gov/nchs/nhanes.htm]. Accessed 11 Nov 2016.

[18] Fishman EI, Stokes A, Preston SH (2014). The dynamics of diabetes among birth cohorts in the U.S. Diabetes Care.

[19] Holmberg SD, Spradling PR, Moorman AC, Denniston MM (2013). Hepatitis C in the United States. N Engl J Med.

[20] Centers for Disease Control and Prevention (2011). National Health and nutrition examination survey: laboratory procedures manual.

[21] Recommendations for Prevention and Control of Hepatitis C Virus (HCV) Infection and HCV-Related Chronic Disease [http://www.cdc.gov/hepatitis/hcv/management.htm]. Accessed 11 Nov 2016.

[22] Population Estimates [https://www.census.gov/programs-surveys/popest/data/data-sets.html]. Accessed 11 Nov 2016.

[23] Ruggles S, Genadek K, Goeken R, Grover J, Sobek M (2015). Integrated public use microdata series: version 6.0.

[24] American Community Survey (ACS) [https://www.census.gov/programs-surveys/acs/]. Accessed 11 Nov 2016.

[25] National Health and Nutrition Examination Survey: Analytic Guidelines, 2011–2012 [https://wwwn.cdc.gov/nchs/data/nhanes/2011-2012/analytic_guidelines_11_12.pdf]. Accessed 11 Nov 2016.

[26] Rao JNK, Molina I. Small Area Estimation. Hoboken: Wiley; 2015.

[27] Kleinbaum DG, Kupper LL, Nizam A, Rosenberg ES (2013). Applied regression analysis and other multivariable methods: Nelson education.

[28] Klein RJ, Proctor SE, Boudreault MA, Turczyn KM. Healthy people 2010 criteria for data suppression. Healthy People 2010 Stat Notes. 2002;(24):1–12. 12117004

[29] Lansky A, Finlayson T, Johnson C, Holtzman D, Wejnert C, Mitsch A, Gust D, Chen R, Mizuno Y, Crepaz N (2014). Estimating the number of persons who inject drugs in the United States by meta-analysis to calculate national rates of HIV and hepatitis C virus infections. PLoS One.

[30] Esmaeili A, Mirzazadeh A, Carter GM, Esmaeili A, Hajarizadeh B, Sacks HS, Page KA (2017). Higher incidence of HCV in females compared to males who inject drugs: a systematic review and meta-analysis. J Viral Hepat.

[31] Page K, Hahn JA, Evans J, Shiboski S, Lum P, Delwart E, Tobler L, Andrews W, Avanesyan L, Cooper S (2009). Acute hepatitis C virus infection in young adult injection drug users: a prospective study of incident infection, resolution, and reinfection. J Infect Dis.

[32] Varan AK, Mercer DW, Stein MS, Spaulding AC (2014). Hepatitis C seroprevalence among prison inmates since 2001: still high but declining. Public Health Reports (Washington, DC : 1974).

[33] Thomas DL, Astemborski J, Rai RM, Anania FA, Schaeffer M, Galai N, Nolt K, Nelson KE, Strathdee SA, Johnson L (2000). The natural history of hepatitis C virus infection: host, viral, and environmental factors. JAMA.

[34] Villano SA, Vlahov D, Nelson KE, Cohn S, Thomas DL (1999). Persistence of viremia and the importance of long-term follow-up after acute hepatitis C infection. Hepatology (Baltimore, Md).

[35] Sarkar M, Bacchetti P, Tien P, Mileti E, French AL, Edlin BR, Keller M, Seaberg E, Nowicki MJ, Young M (2013). Racial/ethnic differences in spontaneous HCV clearance in HIV infected and uninfected women. Dig Dis Sci.

[36] Bourgi K, Brar I, Baker-Genaw K (2016). Health disparities in hepatitis C screening and linkage to Care at an Integrated Health System in Southeast Michigan. PLoS One.

[37] Melia MT, Muir AJ, McCone J, Shiffman ML, King JW, Herrine SK, Galler GW, Bloomer JR, Nunes FA, Brown KA (2011). Racial differences in hepatitis C treatment eligibility. Hepatology (Baltimore, Md).

[38] Vutien P, Hoang J, Brooks L, Jr., Nguyen NH, Nguyen MH. Racial disparities in treatment rates for chronic hepatitis C: analysis of a population-based cohort of 73,665 patients in the United States. Medicine 2016, 95(22):e3719. 10.1097/MD.0000000000003719 PMC4900706 27258498

[39] Schaeffer S, Khalili M (2015). Reasons for HCV non-treatment in underserved African Americans: implications for treatment with new therapeutics. Ann Hepatol.

[40] Fried MW, Shiffman ML, Reddy KR, Smith C, Marinos G, Goncales FL, Haussinger D, Diago M, Carosi G, Dhumeaux D (2002). Peginterferon alfa-2a plus ribavirin for chronic hepatitis C virus infection. N Engl J Med.

[41] Manns MP, McHutchison JG, Gordon SC, Rustgi VK, Shiffman M, Reindollar R, Goodman ZD, Koury K, Ling M, Albrecht JK (2001). Peginterferon alfa-2b plus ribavirin compared with interferon alfa-2b plus ribavirin for initial treatment of chronic hepatitis C: a randomised trial. Lancet (London England).

[42] Khokhar OS, Lewis JH (2007). Reasons why patients infected with chronic hepatitis C virus choose to defer treatment: do they alter their decision with time?. Dig Dis Sci.

[43] Borum ML, Igiehon E, Shafa S (2009). African Americans may differ in their reasons for declining hepatitis C therapy compared to non-African Americans. Dig Dis Sci.

[44] Rousseau CM, Ioannou GN, Todd-Stenberg JA, Sloan KL, Larson MF, Forsberg CW, Dominitz JA (2008). Racial differences in the evaluation and treatment of hepatitis C among veterans: a retrospective cohort study. Am J Public Health.

[45] Kanwal F, Kramer JR, El-Serag HB, Frayne S, Clark J, Cao Y, Taylor T, Smith D, White D, Asch SM (2016). Race and gender differences in the use of direct acting antiviral agents for hepatitis C virus. Clin Infect Dis.

[46] Hepatitis C Disproportionately Affects the African American Community [https://www.cdc.gov/hepatitis/blackhistmnth-hepc.htm]. Accessed 11 Nov 2016.

[47] Edlin BR, Eckhardt BJ, Shu MA, Holmberg SD, Swan T (2015). Toward a more accurate estimate of the prevalence of hepatitis C in the United States. Hepatology (Baltimore, Md).

