# Supplementary material for: Estimates of state-level chronic hepatitis C virus infection, stratified by race and sex, United States, 2010
Source: BMC Infect Dis. 2018 May 16;18:224. doi: 10.1186/s12879-018-3133-6 (PMC5956841; doi:10.1186/s12879-018-3133-6)
Supplement: Supplementary file 1 — Tables S1-S5 that display population counts used as denominators in rate calculations and HCV antibody prevalence by the same race and sex groups as the primary results. (PDF 1163 kb) [file 12879_2018_3133_MOESM1_ESM.pdf]

Estimates of State-level Chronic Hepatitis C Virus Infection, Stratified by Race and Sex, United States, 2010.

Eric W. Hall MPH[1], Eli S. Rosenberg PhD[2], Patrick S. Sullivan DVM PhD[1]

[1] Department of Epidemiology, Emory University Rollins School of Public Health

[2] Department of Epidemiology and Biostatistics, University at Albany School of Public Health

Additional file 1

Table of Contents

Table S1. Estimated Totals of the Non-Institutionalized Population from 2006-2010 5-year American Community Survey Microdata[a] by Sex or Race, US States and District of Columbia, 2010..........2

Table S2. Estimated Total and Prevalence Rate with HCV Antibodies by Sex, US States and District of Columbia, 2010 4

Table S3. Estimated Total and Prevalence Rate with Hepatitis C Antibodies among non-Hispanic White Persons, US States and District of Columbia, 2010 6

Table S4. Estimated Total and Prevalence Rate with Hepatitis C Antibodies among non-Hispanic Black Persons, US States and District of Columbia, 2010 8

Table S5. Estimated Total and Prevalence Rate with Hepatitis C Antibodies among Hispanic Persons, US States and District of Columbia, 2010. 10

References 11

Table S1. Estimated Totals of the Non-Institutionalized Population from 2006-2010 5-year American Community Survey Microdata[a] by Sex or Race, US States and District of Columbia, 2010.

	Sex		Race/Ethnicity		
**State**	**Male**	**Female**	**Non-Hispanic White**	**Non-Hispanic Black**	**Hispanic**
ALABAMA	1,662,073	1,853,907	2,471,932	850,996	193,052
ALASKA	259,368	240,881	342,389	15,537	142,323
ARIZONA	2,219,279	2,334,087	2,942,912	153,949	1,456,505
ARKANSAS	1,017,800	1,103,662	1,661,719	295,347	164,396
CALIFORNIA	13,154,833	13,762,680	12,278,519	1,534,784	13,104,210
COLORADO	1,790,568	1,835,926	2,707,889	124,263	794,342
CONNECTICUT	1,272,230	1,396,804	2,015,214	226,849	426,971
DELAWARE	315,347	348,675	466,715	124,822	72,485
DISTRICT OF COLUMBIA	219,110	254,875	177,202	229,553	67,230
FLORIDA	6,816,416	7,435,690	8,923,115	1,881,139	3,447,852
GEORGIA	3,262,362	3,603,438	4,134,067	1,929,984	801,749
HAWAII	506,818	515,603	264,111	15,345	742,965
IDAHO	539,030	552,433	953,491	5,108	132,864
ILLINOIS	4,539,711	4,892,239	6,404,947	1,253,504	1,773,499
INDIANA	2,276,988	2,441,853	3,999,116	371,876	347,849
IOWA	1,097,495	1,153,519	2,061,812	49,706	139,496
KANSAS	1,001,780	1,051,830	1,689,499	106,295	257,816
KENTUCKY	1,536,992	1,661,361	2,838,803	220,891	138,659
LOUISIANA	1,537,871	1,701,668	2,086,584	933,727	219,228
MAINE	498,391	535,751	991,331	7,377	35,434
MARYLAND	2,014,651	2,254,229	2,496,839	1,172,211	599,830
MASSACHUSETTS	2,360,563	2,610,016	3,968,108	273,977	728,494
MICHIGAN	3,566,768	3,853,711	5,912,830	949,969	557,680
MINNESOTA	1,915,520	1,989,047	3,393,100	155,215	356,252
MISSISSIPPI	1,002,069	1,129,150	1,320,365	723,858	86,996
MISSOURI	2,110,481	2,294,931	3,685,051	451,352	269,009
MONTANA	366,611	373,206	668,480	2,525	68,812
NEBRASKA	647,280	676,058	1,141,107	51,209	131,022
NEVADA	976,414	975,300	1,182,391	138,371	630,952
NEW HAMPSHIRE	489,881	515,158	943,859	9,027	52,153
NEW JERSEY	3,114,840	3,427,012	4,127,708	787,855	1,626,289
NEW MEXICO	713,682	763,265	683,617	25,701	767,629
NEW YORK	6,913,589	7,703,021	8,983,051	2,005,501	3,628,058
NORTH CAROLINA	3,298,754	3,623,653	4,806,316	1,379,563	736,528
NORTH DAKOTA	252,199	250,210	459,639	4,672	38,098
OHIO	4,101,826	4,480,273	7,207,183	929,494	445,422
OKLAHOMA	1,304,473	1,395,408	1,995,990	175,859	528,032

OREGON	1,394,819	1,467,707	2,373,217	42,473	446,836
PENNSYLVANIA	4,570,611	5,027,703	7,965,340	883,212	749,762
RHODE ISLAND	384,752	427,032	656,047	37,189	118,548
SOUTH CAROLINA	1,603,092	1,777,286	2,282,134	880,565	217,679
SOUTH DAKOTA	288,987	297,537	520,846	4,642	61,036
TENNESSEE	2,225,382	2,440,040	3,678,866	701,534	285,022
TEXAS	8,363,843	8,905,195	8,787,032	1,902,726	6,579,280
UTAH	891,862	912,575	1,505,769	14,853	283,815
VERMONT	236,618	249,805	464,243	3,439	18,741
VIRGINIA	2,837,688	3,062,849	4,030,387	1,068,759	801,391
WASHINGTON	2,429,478	2,515,855	3,818,362	153,775	973,196
WEST VIRGINIA	687,995	737,092	1,346,333	39,847	38,907
WISCONSIN	2,060,258	2,159,687	3,678,303	210,865	330,777
WYOMING	206,493	202,489	361,081	2,816	45,085
U.S. STATES & WASHINGTON D.C.	108,855,941	117,173,382	153,854,961	25,514,106	46,660,256

[a]The 2006-2010 5-year sample is a 5% random sample of the national population comprised of all persons from the 1% American Community Survey samples from 2006, 2007, 2008, 2009 and 2010. Individuals from institutionalized group quarters have been removed (1, 2).

Table S2. Estimated Total and Prevalence Rate with HCV Antibodies by Sex, US States and District of Columbia, 2010

	Female						Male								
	Anti-HCV Prevalence Rate (per 100)			Total Persons with Anti-HCV			Anti-HCV Prevalence Rate (per 100)			Total Persons with Anti-HCV			Rate Ratio (ref=Female)		
**State**	**Rate**	**(95% CI)**		**n**	**(95% CI)**		**Rate**	**(95% CI)**		**n**	**(95% CI)**		**Ratio**	**(95% CI)**	
ALABAMA	1.09	(0.90	1.38)	20,233	(16,727	25,610)	1.86	(1.63	2.17)	30,937	(27,039	36,081)	1.71	(1.30	2.19)
ALASKA	2.22	(1.65	3.24)	5,341	(3,963	7,816)	2.13	(1.78	2.69)	5,531	(4,614	6,969)	0.96	(0.65	1.39)
ARIZONA	1.30	(1.06	1.67)	30,306	(24,785	38,992)	2.55	(2.23	2.98)	56,686	(49,468	66,146)	1.97	(1.48	2.55)
ARKANSAS	1.18	(0.94	1.56)	13,021	(10,351	17,243)	2.28	(1.96	2.71)	23,255	(19,901	27,552)	1.94	(1.41	2.58)
CALIFORNIA	1.69	(1.43	2.12)	233,188	(196,641	292,405)	2.97	(2.64	3.42)	390,773	(347,416	450,511)	1.75	(1.35	2.20)
COLORADO	1.29	(1.06	1.64)	23,599	(19,419	30,189)	2.30	(2.02	2.68)	41,240	(36,208	47,962)	1.79	(1.35	2.30)
CONNECTICUT	0.92	(0.77	1.17)	12,867	(10,802	16,276)	1.85	(1.63	2.14)	23,475	(20,730	27,221)	2.00	(1.54	2.52)
DELAWARE	1.43	(1.16	1.88)	4,977	(4,043	6,558)	2.61	(2.25	3.13)	8,216	(7,096	9,868)	1.83	(1.34	2.41)
DISTRICT OF COLUMBIA	3.06	(2.44	3.96)	7,790	(6,216	10,091)	3.93	(3.32	4.81)	8,621	(7,284	10,529)	1.29	(0.94	1.74)
FLORIDA	1.21	(1.00	1.53)	89,662	(74,422	113,440)	2.21	(1.94	2.56)	150,765	(132,145	174,689)	1.83	(1.41	2.34)
GEORGIA	0.86	(0.72	1.07)	31,012	(25,969	38,465)	1.57	(1.38	1.81)	51,222	(45,034	59,195)	1.82	(1.42	2.30)
HAWAII	0.80	(0.63	1.14)	4,138	(3,232	5,876)	1.99	(1.71	2.38)	10,085	(8,687	12,061)	2.48	(1.69	3.38)
IDAHO	1.14	(0.87	1.61)	6,315	(4,787	8,881)	1.76	(1.48	2.15)	9,467	(7,979	11,577)	1.54	(1.05	2.18)
ILLINOIS	0.56	(0.48	0.70)	27,410	(23,298	34,045)	0.90	(0.80	1.04)	41,034	(36,311	47,240)	1.61	(1.26	2.01)
INDIANA	0.91	(0.74	1.17)	22,192	(18,051	28,498)	1.56	(1.35	1.83)	35,469	(30,784	41,606)	1.71	(1.29	2.25)
IOWA	0.70	(0.54	0.96)	8,031	(6,179	11,035)	1.45	(1.24	1.75)	15,965	(13,642	19,196)	2.09	(1.47	2.89)
KANSAS	1.01	(0.80	1.35)	10,584	(8,405	14,169)	1.86	(1.59	2.21)	18,610	(15,954	22,142)	1.85	(1.34	2.48)
KENTUCKY	1.17	(0.92	1.55)	19,503	(15,346	25,804)	2.12	(1.81	2.55)	32,651	(27,786	39,152)	1.81	(1.31	2.45)
LOUISIANA	1.67	(1.41	2.06)	28,440	(23,977	35,034)	2.92	(2.58	3.37)	44,881	(39,736	51,797)	1.75	(1.38	2.19)
MAINE	0.58	(0.42	0.89)	3,121	(2,239	4,771)	1.59	(1.32	1.99)	7,947	(6,571	9,918)	2.74	(1.71	4.10)
MARYLAND	1.54	(1.31	1.87)	34,645	(29,460	42,188)	2.27	(2.01	2.60)	45,665	(40,560	52,363)	1.47	(1.17	1.82)
MASSACHUSETTS	1.04	(0.85	1.33)	27,173	(22,289	34,714)	1.92	(1.68	2.23)	45,262	(39,766	52,662)	1.84	(1.39	2.37)
MICHIGAN	1.00	(0.84	1.24)	38,540	(32,554	47,629)	1.75	(1.54	2.01)	62,259	(55,040	71,638)	1.75	(1.37	2.17)
MINNESOTA	0.69	(0.57	0.89)	13,713	(11,270	17,643)	1.38	(1.21	1.61)	26,473	(23,212	30,846)	2.00	(1.51	2.58)
MISSISSIPPI	1.15	(0.94	1.48)	12,960	(10,605	16,675)	2.14	(1.85	2.53)	21,429	(18,567	25,364)	1.86	(1.39	2.42)
MISSOURI	1.20	(0.98	1.52)	27,472	(22,575	34,848)	2.24	(1.95	2.61)	47,257	(41,244	55,162)	1.87	(1.42	2.41)
MONTANA	1.65	(1.25	2.31)	6,147	(4,672	8,611)	2.13	(1.79	2.62)	7,794	(6,566	9,611)	1.29	(0.88	1.83)
NEBRASKA	0.89	(0.70	1.21)	6,002	(4,745	8,149)	1.50	(1.28	1.81)	9,691	(8,314	11,691)	1.69	(1.20	2.28)

NEVADA	1.18	(0.94	1.55)	11,466	(9,187	15,155)	2.48	(2.14	2.93)	24,246	(20,938	28,629)	2.11	(1.54	2.79)
NEW HAMPSHIRE	0.70	(0.50	1.05)	3,587	(2,588	5,406)	1.49	(1.24	1.87)	7,320	(6,072	9,141)	2.15	(1.36	3.22)
NEW JERSEY	1.03	(0.88	1.27)	35,219	(29,985	43,474)	1.76	(1.56	2.01)	54,728	(48,633	62,755)	1.71	(1.35	2.12)
NEW MEXICO	1.87	(1.50	2.50)	14,305	(11,461	19,106)	3.83	(3.27	4.67)	27,355	(23,371	33,296)	2.05	(1.47	2.75)
NEW YORK	1.12	(0.97	1.37)	86,482	(74,843	105,194)	2.00	(1.80	2.28)	138,393	(124,610	157,335)	1.78	(1.42	2.17)
NORTH CAROLINA	1.10	(0.92	1.37)	40,001	(33,417	49,666)	2.21	(1.96	2.55)	73,062	(64,529	84,054)	2.01	(1.56	2.52)
NORTH DAKOTA	0.53*	(0.37	0.92)	1,319*	(915	2,304)	1.08	(0.88	1.45)	2,725	(2,224	3,651)	2.05*	(1.14	3.26)
OHIO	0.95	(0.80	1.19)	42,581	(35,631	53,108)	1.82	(1.60	2.09)	74,487	(65,523	85,825)	1.91	(1.49	2.41)
OKLAHOMA	2.73	(2.18	3.55)	38,057	(30,429	49,499)	3.92	(3.39	4.60)	51,118	(44,186	59,967)	1.44	(1.07	1.90)
OREGON	2.29	(1.79	3.05)	33,605	(26,280	44,727)	3.88	(3.32	4.60)	54,170	(46,369	64,166)	1.70	(1.23	2.31)
PENNSYLVANIA	0.98	(0.82	1.21)	49,122	(41,395	60,770)	1.94	(1.72	2.24)	88,889	(78,595	102,231)	1.99	(1.56	2.48)
RHODE ISLAND	1.65	(1.31	2.22)	7,027	(5,596	9,459)	2.66	(2.29	3.18)	10,223	(8,818	12,232)	1.61	(1.16	2.17)
SOUTH CAROLINA	1.26	(1.04	1.58)	22,352	(18,537	28,127)	2.36	(2.07	2.74)	37,832	(33,178	43,851)	1.88	(1.45	2.40)
SOUTH DAKOTA	0.71	(0.52	1.13)	2,116	(1,534	3,348)	1.27	(1.05	1.65)	3,680	(3,040	4,765)	1.79	(1.10	2.70)
TENNESSEE	1.77	(1.44	2.26)	43,255	(35,130	55,063)	3.38	(2.92	3.96)	75,138	(65,086	88,137)	1.90	(1.44	2.49)
TEXAS	1.52	(1.30	1.88)	135,504	(115,880	167,080)	2.76	(2.46	3.17)	231,081	(205,461	265,116)	1.82	(1.43	2.25)
UTAH	0.69	(0.53	0.95)	6,275	(4,875	8,705)	1.23	(1.04	1.49)	10,927	(9,290	13,248)	1.78	(1.24	2.47)
VERMONT	0.96	(0.67	1.54)	2,400	(1,671	3,837)	2.00	(1.61	2.57)	4,726	(3,820	6,084)	2.08*	(1.25	3.22)
VIRGINIA	0.74	(0.62	0.92)	22,553	(18,960	28,052)	1.50	(1.33	1.73)	42,570	(37,602	49,035)	2.04	(1.60	2.55)
WASHINGTON	1.69	(1.35	2.20)	42,537	(34,046	55,267)	2.96	(2.57	3.47)	72,024	(62,446	84,274)	1.75	(1.30	2.33)
WEST VIRGINIA	1.11	(0.83	1.57)	8,174	(6,138	11,586)	2.20	(1.84	2.71)	15,164	(12,673	18,622)	1.99	(1.35	2.83)
WISCONSIN	0.49	(0.40	0.64)	10,658	(8,743	13,767)	0.97	(0.84	1.13)	19,893	(17,356	23,323)	1.96	(1.46	2.53)
WYOMING	1.56	(1.13	2.41)	3,159	(2,285	4,876)	2.10	(1.70	2.73)	4,342	(3,513	5,640)	1.35	(0.84	2.04)
U.S. STATES & WASHINGTON D.C.	1.22	(1.04	1.51)	1,430,132	(1,215,849	1,774,777)	2.20	(1.96	2.52)	2,396,753	(2,132,310	2,747,709)	1.80	(1.42	2.24)

Abbreviations: CI, confidence interval; anti-HCV, hepatitis C virus antibody

*Coefficient of variation is ≥23%; estimate is unreliable.

Table S3. Estimated Total and Prevalence Rate with Hepatitis C Antibodies among non-Hispanic White Persons, US States and District of Columbia, 2010

	anti-HCV Prevalence Rate (per 100)			Total Persons with anti-HCV		
**State**	**Rate**	**(95% CI)**		**n**	**(95% CI)**	
ALABAMA	1.46	(1.26	1.74)	36,129	(31,224	43,001)
ALASKA	2.52	(2.07	3.23)	8,631	(7,104	11,056)
ARIZONA	2.10	(1.82	2.47)	61,726	(53,560	72,666)
ARKANSAS	1.80	(1.54	2.15)	29,842	(25,607	35,705)
CALIFORNIA	2.92	(2.54	3.41)	358,117	(311,925	419,237)
COLORADO	1.58	(1.36	1.87)	42,887	(36,920	50,689)
CONNECTICUT	1.11	(0.95	1.32)	22,340	(19,240	26,653)
DELAWARE	1.69	(1.42	2.10)	7,865	(6,627	9,795)
DISTRICT OF COLUMBIA	0.69	(0.55	0.96)	1,224	(967	1,696)
FLORIDA	1.99	(1.73	2.34)	177,752	(154,594	208,475)
GEORGIA	1.28	(1.11	1.51)	52,928	(45,795	62,425)
HAWAII	3.43	(2.91	4.20)	9,048	(7,674	11,087)
IDAHO	1.46	(1.23	1.80)	13,918	(11,770	17,130)
ILLINOIS	0.68	(0.59	0.80)	43,322	(37,525	51,292)
INDIANA	1.13	(0.97	1.34)	45,056	(38,963	53,451)
IOWA	1.01	(0.86	1.21)	20,725	(17,747	25,028)
KANSAS	1.39	(1.19	1.68)	23,541	(20,123	28,351)
KENTUCKY	1.58	(1.35	1.89)	44,916	(38,414	53,690)
LOUISIANA	2.03	(1.76	2.41)	42,411	(36,679	50,204)
MAINE	1.07	(0.91	1.33)	10,606	(8,979	13,163)
MARYLAND	1.46	(1.26	1.74)	36,527	(31,450	43,426)
MASSACHUSETTS	1.34	(1.15	1.58)	53,013	(45,815	62,671)
MICHIGAN	1.07	(0.93	1.26)	63,316	(54,783	74,608)
MINNESOTA	0.83	(0.71	0.98)	28,013	(24,106	33,262)
MISSISSIPPI	1.88	(1.61	2.26)	24,834	(21,288	29,805)
MISSOURI	1.53	(1.33	1.81)	56,542	(49,022	66,784)
MONTANA	1.73	(1.44	2.15)	11,532	(9,615	14,369)
NEBRASKA	1.03	(0.87	1.27)	11,780	(9,923	14,492)
NEVADA	2.36	(2.02	2.81)	27,871	(23,915	33,221)
NEW HAMPSHIRE	1.09	(0.92	1.36)	10,308	(8,695	12,857)
NEW JERSEY	1.34	(1.16	1.58)	55,382	(48,083	65,293)
NEW MEXICO	2.65	(2.28	3.15)	18,139	(15,594	21,551)
NEW YORK	1.12	(0.98	1.32)	100,870	(87,864	118,215)
NORTH CAROLINA	1.53	(1.32	1.79)	73,366	(63,571	85,936)
NORTH DAKOTA	0.71	(0.57	0.98)	3,253	(2,606	4,525)
OHIO	1.11	(0.97	1.31)	80,145	(69,659	94,440)
OKLAHOMA	3.70	(3.19	4.39)	73,890	(63,706	87,719)

OREGON	3.30	(2.85	3.91)	78,348	(67,582	92,724)
PENNSYLVANIA	1.14	(0.99	1.34)	90,679	(78,809	106,418)
RHODE ISLAND	1.93	(1.63	2.37)	12,665	(10,678	15,526)
SOUTH CAROLINA	1.78	(1.54	2.11)	40,703	(35,115	48,218)
SOUTH DAKOTA	0.85	(0.69	1.14)	4,451	(3,600	5,933)
TENNESSEE	2.56	(2.22	3.03)	94,360	(81,826	111,377)
TEXAS	2.35	(2.05	2.76)	206,810	(180,089	242,120)
UTAH	0.94	(0.80	1.15)	14,121	(11,990	17,260)
VERMONT	1.47	(1.22	1.90)	6,826	(5,676	8,800)
VIRGINIA	1.00	(0.87	1.18)	40,295	(34,884	47,569)
WASHINGTON	2.44	(2.11	2.88)	93,212	(80,516	110,023)
WEST VIRGINIA	1.60	(1.35	1.95)	21,511	(18,202	26,250)
WISCONSIN	0.59	(0.51	0.71)	21,815	(18,771	26,125)
WYOMING	1.80	(1.47	2.36)	6,503	(5,301	8,535)
U.S. STATES & WASHINGTON D.C.	1.63	(1.43	1.91)	2,514,068	(2,197,215	2,942,026)

Abbreviations: CI, confidence interval; anti-HCV, hepatitis C virus antibody

*Coefficient of variation is ≥23%; estimate is unreliable.

Table S4. Estimated Total and Prevalence Rate with Hepatitis C Antibodies among non-Hispanic Black Persons, US States and District of Columbia, 2010

	anti-HCV Prevalence Rate (per 100)			Total Persons with anti-HCV			Rate Ratio (ref=white)		
**State**	**Rate**	**(95% CI)**		**n**	**(95% CI)**		**Ratio**	**(95% CI)**	
ALABAMA	1.69	(1.47	2.02)	14,420	(12,504	17,159)	1.16	(0.93	1.45)
ALASKA	2.77	(2.02	4.39)	431	(314	682)	1.10	(0.80	1.68)
ARIZONA	2.93	(2.49	3.54)	4,504	(3,826	5,447)	1.39	(1.11	1.77)
ARKANSAS	1.99	(1.71	2.41)	5,886	(5,045	7,118)	1.11	(0.88	1.41)
CALIFORNIA	5.05	(4.42	5.89)	77,497	(67,837	90,369)	1.73	(1.41	2.13)
COLORADO	4.52	(3.88	5.39)	5,620	(4,820	6,698)	2.86	(2.27	3.59)
CONNECTICUT	3.21	(2.76	3.84)	7,292	(6,263	8,714)	2.90	(2.30	3.65)
DELAWARE	3.90	(3.31	4.79)	4,871	(4,130	5,984)	2.32	(1.76	3.02)
DISTRICT OF COLUMBIA	6.48	(5.59	7.78)	14,881	(12,826	17,858)	9.38	(6.59	12.63)
FLORIDA	1.89	(1.65	2.21)	35,484	(30,967	41,637)	0.95	(0.77	1.17)
GEORGIA	1.42	(1.23	1.67)	27,355	(23,761	32,215)	1.11	(0.89	1.38)
HAWAII	2.76	(2.09	3.95)	423	(321	606)	0.80	(0.57	1.18)
IDAHO	1.67*	(0.94	4.16)	85*	(48	212)	1.14*	(0.61	2.86)
ILLINOIS	1.58	(1.38	1.86)	19,824	(17,302	23,305)	2.34	(1.89	2.89)
INDIANA	2.95	(2.56	3.51)	10,988	(9,534	13,057)	2.62	(2.10	3.28)
IOWA	3.97	(3.29	4.98)	1,973	(1,634	2,477)	3.95	(3.04	5.20)
KANSAS	3.18	(2.70	3.88)	3,384	(2,867	4,125)	2.28	(1.79	2.93)
KENTUCKY	2.94	(2.51	3.58)	6,503	(5,547	7,900)	1.86	(1.47	2.38)
LOUISIANA	3.16	(2.76	3.73)	29,527	(25,756	34,788)	1.56	(1.26	1.93)
MAINE	2.76*	(1.87	4.69)	203*	(138	346)	2.58*	(1.63	4.46)
MARYLAND	3.54	(3.09	4.16)	41,527	(36,169	48,777)	2.42	(1.94	3.01)
MASSACHUSETTS	3.44	(2.98	4.11)	9,423	(8,151	11,252)	2.57	(2.06	3.22)
MICHIGAN	3.46	(3.02	4.07)	32,902	(28,666	38,644)	3.23	(2.61	4.00)
MINNESOTA	4.96	(4.24	5.95)	7,696	(6,587	9,241)	6.01	(4.76	7.58)
MISSISSIPPI	1.25	(1.07	1.50)	9,032	(7,776	10,851)	0.66	(0.52	0.84)
MISSOURI	3.58	(3.12	4.23)	16,180	(14,088	19,069)	2.34	(1.88	2.91)
MONTANA	5.93*	(3.89	10.70)	150*	(98	270)	3.44*	(2.12	6.35)
NEBRASKA	5.02	(4.20	6.30)	2,573	(2,152	3,225)	4.87	(3.71	6.46)
NEVADA	2.32	(1.96	2.81)	3,208	(2,717	3,894)	0.98	(0.77	1.25)
NEW HAMPSHIRE	2.53*	(1.71	4.24)	228*	(154	383)	2.31*	(1.47	3.92)
NEW JERSEY	2.99	(2.62	3.51)	23,581	(20,622	27,651)	2.23	(1.81	2.75)
NEW MEXICO	3.17	(2.55	4.16)	815	(656	1,070)	1.20	(0.91	1.62)
NEW YORK	3.39	(2.97	3.95)	67,976	(59,482	79,184)	3.02	(2.45	3.71)
NORTH CAROLINA	2.60	(2.26	3.06)	35,804	(31,203	42,176)	1.70	(1.37	2.10)
NORTH DAKOTA	0.94*	(0.50	2.94)	44*	(24	137)	1.33*	(0.65	4.16)
OHIO	3.66	(3.20	4.29)	34,048	(29,781	39,894)	3.29	(2.67	4.06)
OKLAHOMA	3.17	(2.72	3.80)	5,568	(4,775	6,674)	0.86	(0.68	1.08)

OREGON	6.00	(5.06	7.33)	2,549	(2,149	3,114)	1.82	(1.43	2.33)
PENNSYLVANIA	4.27	(3.73	5.01)	37,683	(32,918	44,249)	3.75	(3.04	4.62)
RHODE ISLAND	6.46	(5.41	8.07)	2,403	(2,014	3,001)	3.35	(2.55	4.39)
SOUTH CAROLINA	2.07	(1.80	2.43)	18,209	(15,810	21,420)	1.16	(0.93	1.44)
SOUTH DAKOTA	3.69*	(2.46	6.68)	171*	(114	310)	4.32*	(2.61	8.07)
TENNESSEE	3.20	(2.77	3.78)	22,414	(19,416	26,538)	1.25	(1.00	1.56)
TEXAS	3.75	(3.27	4.42)	71,381	(62,222	83,999)	1.59	(1.29	1.97)
UTAH	3.14	(2.34	4.54)	467	(347	674)	3.35	(2.34	4.97)
VERMONT	3.14*	(2.05	5.86)	108*	(70	202)	2.13*	(1.29	4.04)
VIRGINIA	2.03	(1.77	2.39)	21,701	(18,871	25,518)	2.03	(1.64	2.51)
WASHINGTON	5.38	(4.65	6.38)	8,266	(7,144	9,806)	2.20	(1.77	2.75)
WEST VIRGINIA	4.32	(3.58	5.42)	1,719	(1,428	2,159)	2.70	(2.05	3.58)
WISCONSIN	3.11	(2.67	3.73)	6,557	(5,628	7,868)	5.24	(4.15	6.62)
WYOMING	2.23*	(1.34	5.27)	63*	(38	148)	1.24*	(0.69	2.95)
U.S. STATES & WASHINGTON D.C.	2.96	(2.60	3.46)	755,596	(663,906	882,217)	1.81	(1.48	2.22)

Abbreviations: CI, confidence interval; anti-HCV, hepatitis C virus antibody

*Coefficient of variation is ≥23%; estimate is unreliable.

Table S5. Estimated Total and Prevalence Rate with Hepatitis C Antibodies among Hispanic Persons, US States and District of Columbia, 2010.

	anti-HCV Prevalence Rate (per 100)			Total Persons with anti-HCV			Rate Ratio (ref=white)		
**State**	**Rate**	**(95% CI)**		**n**	**(95% CI)**		**Ratio**	**(95% CI)**	
ALABAMA	0.32	(0.25	0.45)	620	(479	872)	0.22	(0.16	0.32)
ALASKA	1.27	(0.97	1.86)	1,810	(1,378	2,645)	0.50	(0.38	0.72)
ARIZONA	1.43	(1.17	1.83)	20,762	(17,062	26,667)	0.68	(0.53	0.91)
ARKANSAS	0.33	(0.25	0.48)	547	(417	784)	0.19	(0.13	0.27)
CALIFORNIA	1.44	(1.19	1.84)	188,347	(156,015	241,748)	0.49	(0.39	0.66)
COLORADO	2.06	(1.70	2.64)	16,332	(13,478	20,931)	1.30	(1.01	1.74)
CONNECTICUT	1.57	(1.28	2.06)	6,710	(5,451	8,802)	1.42	(1.08	1.92)
DELAWARE	0.63	(0.47	0.95)	457	(341	692)	0.37	(0.26	0.58)
DISTRICT OF COLUMBIA	0.45	(0.34	0.69)	305	(227	467)	0.66*	(0.42	1.06)
FLORIDA	0.79	(0.65	1.02)	27,191	(22,477	35,041)	0.40	(0.31	0.53)
GEORGIA	0.24	(0.19	0.32)	1,950	(1,557	2,603)	0.19	(0.14	0.26)
HAWAII	0.64	(0.52	0.86)	4,752	(3,831	6,382)	0.19	(0.14	0.26)
IDAHO	1.34	(1.07	1.82)	1,779	(1,421	2,421)	0.92	(0.68	1.31)
ILLINOIS	0.30	(0.24	0.40)	5,298	(4,313	7,028)	0.44	(0.34	0.60)
INDIANA	0.46	(0.37	0.63)	1,616	(1,298	2,178)	0.41	(0.31	0.57)
IOWA	0.93	(0.73	1.30)	1,298	(1,014	1,809)	0.93	(0.68	1.34)
KANSAS	0.88	(0.70	1.18)	2,269	(1,811	3,053)	0.63	(0.47	0.88)
KENTUCKY	0.53	(0.40	0.77)	734	(554	1,071)	0.33	(0.24	0.50)
LOUISIANA	0.63	(0.51	0.85)	1,382	(1,115	1,857)	0.31	(0.24	0.43)
MAINE	0.73	(0.53	1.14)	259	(186	403)	0.68	(0.46	1.10)
MARYLAND	0.38	(0.30	0.50)	2,257	(1,827	2,996)	0.26	(0.20	0.35)
MASSACHUSETTS	1.37	(1.12	1.79)	9,998	(8,150	13,019)	1.03	(0.79	1.39)
MICHIGAN	0.82	(0.67	1.06)	4,581	(3,749	5,914)	0.77	(0.59	1.03)
MINNESOTA	1.26	(1.02	1.64)	4,477	(3,632	5,852)	1.52	(1.17	2.06)
MISSISSIPPI	0.60	(0.46	0.87)	523	(399	761)	0.32	(0.23	0.47)
MISSOURI	0.75	(0.60	0.98)	2,006	(1,622	2,647)	0.49	(0.37	0.67)
MONTANA	3.28	(2.61	4.47)	2,259	(1,797	3,075)	1.90	(1.40	2.71)
NEBRASKA	1.02	(0.80	1.44)	1,340	(1,045	1,890)	0.99	(0.71	1.45)
NEVADA	0.73	(0.60	0.96)	4,633	(3,775	6,047)	0.31	(0.24	0.42)
NEW HAMPSHIRE	0.71	(0.52	1.07)	371	(274	561)	0.65	(0.45	1.02)
NEW JERSEY	0.68	(0.56	0.87)	10,984	(9,071	14,180)	0.50	(0.39	0.68)
NEW MEXICO	2.96	(2.43	3.84)	22,705	(18,674	29,454)	1.11	(0.86	1.51)
NEW YORK	1.54	(1.28	1.98)	56,029	(46,489	71,958)	1.38	(1.07	1.83)
NORTH CAROLINA	0.53	(0.42	0.70)	3,893	(3,126	5,134)	0.35	(0.27	0.47)
NORTH DAKOTA	1.96	(1.46	3.08)	746	(555	1,172)	2.77*	(1.80	4.51)
OHIO	0.65	(0.53	0.84)	2,875	(2,352	3,729)	0.58	(0.45	0.78)
OKLAHOMA	1.84	(1.51	2.38)	9,718	(7,973	12,576)	0.50	(0.38	0.67)

OREGON	1.54	(1.26	1.97)	6,878	(5,623	8,824)	0.47	(0.36	0.63)
PENNSYLVANIA	1.29	(1.06	1.67)	9,649	(7,910	12,499)	1.13	(0.88	1.51)
RHODE ISLAND	1.84	(1.47	2.51)	2,181	(1,739	2,974)	0.95	(0.70	1.36)
SOUTH CAROLINA	0.58	(0.46	0.79)	1,272	(1,011	1,728)	0.33	(0.25	0.46)
SOUTH DAKOTA	1.92	(1.47	2.83)	1,173	(899	1,725)	2.25	(1.53	3.47)
TENNESSEE	0.57	(0.45	0.78)	1,620	(1,273	2,228)	0.22	(0.17	0.31)
TEXAS	1.34	(1.11	1.72)	88,394	(72,846	113,305)	0.57	(0.45	0.76)
UTAH	0.92	(0.74	1.24)	2,614	(2,108	3,514)	0.98	(0.74	1.38)
VERMONT	1.02	(0.72	1.72)	192	(135	323)	0.70*	(0.45	1.20)
VIRGINIA	0.39	(0.32	0.51)	3,127	(2,543	4,081)	0.39	(0.30	0.53)
WASHINGTON	1.34	(1.11	1.73)	13,083	(10,815	16,870)	0.55	(0.43	0.74)
WEST VIRGINIA	0.28*	(0.18	0.53)	108*	(70	207)	0.17*	(0.11	0.34)
WISCONSIN	0.66	(0.53	0.88)	2,179	(1,747	2,911)	1.11	(0.84	1.53)
WYOMING	2.08	(1.58	3.06)	936	(710	1,382)	1.15	(0.79	1.78)
U.S. STATES & WASHINGTON D.C.	1.19	(0.99	1.53)	557,222	(463,335	714,245)	0.73	(0.57	0.97)

Abbreviations: CI, confidence interval; anti-HCV, hepatitis C virus antibody
*Coefficient of variation is ≥23%; estimate is unreliable.

References

1. United States Census Bureau. American Community Survey (ACS) 2017 [Available from: https://www.census.gov/programs-surveys/acs/.
2. Ruggles S, Genadek K, Goeken R, Grover J, Sobek M. Integrated Public Use Microdata Series: Version 6.0. Minneapolis: University of Minnesota; 2015.
